# Supplementary material for: Regioselective protein oxidative cleavage enabled by enzyme-like recognition of an inorganic metal oxo cluster ligand
Source: Nat Commun. 2023 Jan 30;14:486. doi: 10.1038/s41467-023-36085-z (PMC9887005; doi:10.1038/s41467-023-36085-z)
Supplement: Supplementary file 1 — Supplementary Information [file 41467_2023_36085_MOESM1_ESM.pdf]

Supplementary information

Regioselective Protein Oxidative Cleavage Enabled by Enzyme-like Recognition of an Inorganic Metal Oxo Cluster Ligand

Shorok A. M. Abdelhameed,[a] Francisco de Azambuja,[a] Tamara Vasović, [b] Nada D. Savić, [a] Tanja Cirkovic Velickovic, [b], [c], [d],[e] and Tatjana N. Parac-Vogt* [a]

[a] KU Leuven, Department of Chemistry, Celestijnenlaan 200F, 3001 Leuven, Belgium. [b] Center of Excellence for Molecular Food Sciences & Department of Biochemistry, University of Belgrade - Faculty of Chemistry, Belgrade, Serbia. [c] Ghent University Global Campus, Yeonsu-gu, Incheon, South Korea. [d] Faculty of Bioscience Engineering, Ghent University, Ghent, Belgium. [e] Serbian Academy of Sciences and Arts, Belgrade, Serbia. Email: tatjana.vogt@kuleuven.be

Table of Contents

1. Supplementary Methods ... 2

2. Supplementary discussion ... 4

2.1. Cleavage of HEWL ... 4

2.1.1. Oxidative cleavage ... 4

2.1.2. Hydrolytic cleavage ... 12

2.2. UV-Vis spectroscopy ... 15

2.3. HEWL and Cu^{II}WD interaction studies: ... 16

2.3.1. ^{1}H NMR spectroscopy ... 18

2.3.2. Circular dichroism spectroscopy (CD) ... 19

2.4. Mechanistic studies ... 20

2.5. Identification of oxidative modifications and cleavage sites by nLC-MS/MS. ... 24

3. Supplementary References ... 30

1. Supplementary Methods

General remarks. Unless otherwise noted, reactions were performed without any precautions against air and moisture. The cleavage reactions were performed in 1 mL aqueous solutions where the final concentrations of hen egg white lysozyme (HEWL), $K_8[Cu^{2+}(H_2O)(a_2\text{-}P_2W_{17}O_{61})]$, ($Cu^{II}$WD), or $CuSO_4$ and sodium ascorbate (Asc) were 0.02, 0.05 or 2 and 1 mM, respectively. Stock solutions of HEWL (1 mM), Cu^{II}WD or $CuSO_4$ (10 mM) and Asc (200 mM) were prepared in 10 mM Tris-HCl, pH 7.5, unless otherwise noted. HEWL was purchased from Sigma–Aldrich. Sodium ascorbate was purchased from TCI and $CuSO_4$ was purchased from Fluka Chemika. All chemicals were used without any further purifications. Hydrogen nuclear magnetic resonance (^{1}H NMR) spectra, and phosphorus nuclear magnetic resonance (^{31}P NMR) spectra were recorded on a Bruker Avance 400 spectrometer (400 and 376 MHz, respectively). Chemical shifts (δ) for hydrogens are reported in parts per million (ppm) downfield from tetramethylsilane propionic acid (TMSP-D_4, 0.5 mM, 0 ppm) and are calibrated using the residual solvent peak in the NMR solvent (D_2O: δ = 4.79 ppm). ^{31}P NMR chemical shifts (δ) are reported in ppm upfield from H_3PO_4 25% (0 ppm).

Data analysis. All NMR spectra were analyzed by using TopSpin 4.0.6. CD and UV-Vis measurements were done using Origin 2018b software. SDS-PAGE gels were analyzed by Bio-Rad Image Lab Software Version 6.0.0 3D structure of HEWL (1DPX) was prepared using PyMOL version 2.5.4.

Gel electrophoresis. SDS-Tricine-PAGE (SDS-PAGE) was performed on a stacking gel of 5% (w/v) polyacrylamide gel in 3.0 M Tris-HCl buffer pH 8.45 and a resolving gel 18% (w/v) polyacrylamide in 3.0 M Tris-HCl buffer pH 8.45. Samples (15 µL) were supplemented with 5 µL sample buffer (1M tris-HCl, pH 6.8 (2.25 mL), glycerol (5 mL), SDS (0.5 g), bromophenol blue (5 mg) and 1M dithiothreitol (2.5 mL) and heated at 95 °C for 5 min, followed by loading 10 µL of the resulting solution on the gel. Unstained low range (3.4 to 100 kDa) protein ladder (PL) was used as a molecular mass standard. An OmniPAGE electrophoretic cell was combined with an EV243 power supply (both produced by Consort, Turnhout, Belgium). Experiments were performed at 200 V for 2.0 h. Proteins in SDS-Tricine-PAGE gels were visualized with silver staining and an image of each gel was taken using a GelDoc EZ Imager (Bio-Rad, Hercules, CA). The percentage of the fragment bands compared to the total amount of protein in each lane was determined using the Bio-Rad Image Lab software Version 6.0.0

^{31}P NMR spectroscopy. A 1.5 mL centrifuge tube was charged with 200 µL of 20 mM Cu^{II}WD stock solution, 20 µL of 1 mM HEWL stock solution, 10 µL of 200 mM Asc and 100 µL D_2O and 780 µL buffer – 10 mM tris-HCl (pH 7.4). The final concentration of Cu^{II}WD was 2.0 mM, and of HEWL was 0.02 mM and 2.0 mM of Asc. The reaction mixture was homogenized using a vortex. Next, 500 µL of the reaction mixture was transferred into an NMR tube. The reaction was incubated for 1 day at 60 ºC. Then, the reaction was measured by ^{31}P NMR spectroscopy.

UV-Vis spectroscopy. Quartz cuvettes with 1.0 cm optical path length were used. The cuvette was loaded with 15 µL of 10 mM Cu^{II}WD stock solution, 60 µL of 1 mM HEWL stock solution and 30 µL of 200 mM Asc stock solution. The final concentrations were Cu^{II}WD = 0.05 mM, HEWL = 0.02 mM, and Asc = 1mM. UV-Vis absorption spectra were recorded on a Varian Cary 5000 spectrophotometer.

^{1}H NMR spectroscopy. Solutions containing HEWL (1 mM) in the absence or presence of 10% SDS and 0.1 M DTT or in the presence of Cu^{II}WD (0.05 mM) or Cu^{II}WD (0.05 mM) + Asc (0.05 mM) were prepared in 10.0 mM tris-HCl buffer (pH 7.5, 10% D_2O) at room temperature.

Circular dichroism spectroscopy (CD). Quartz cuvettes with 1.0 mm optical path length were used. The cuvette was loaded with 300 µL of HEWL (0.01 mM) and titrated with Cu^{II}WD in presence and absence of Asc. All Solutions were prepared in 10 mM Tris-HCl buffer pH 7.5 and all measurements were done at 20 □C. CD measurements were performed by using a JASCO J-810 spectropolarimeter. Far-UV wavelength scans were recorded from 180 to 300 nm. All the CD spectra were corrected for the background effect by subtracting the spectrum of the respective buffer solution from the spectrum of the protein.

nLC-MS/MS. After SDS-PAGE and Coomassie Brilliant Blue (CBB) staining and scanning (**Supplementary Fig. 25**), gel bands were excised, and in-gel digested with trypsin using the method described by Shevchenko et al.[32] The proteins were digested with proteomics-grade porcine trypsin at a ratio of 1:30. Peptides from in-gel digestions, were chromatographically separated using an UltiMate™ 3000 RSLC nano-liquid chromatographic system (nLC) (Thermo Fisher Scientific, Bremen, Germany), equipped with a 2-column set up: *i)* a trap column C18, 50 mm, and *ii)* an analytical column PepMap (C18, 15 cm × 75 µm, 3 µm particles, and 100 Å pore size). The mobile phases were (A) water (MS-grade) with 0.1% formic acid and (B) acetonitrile (MS-grade) with 0.1% formic acid. The gradient program was as follows: 0–0.5 min 95% A, 0.5–10 min 95-66% A, 10–15 min -66-0% A, 15–20 min 0% A, 20–23 min 5% A, with flow rate of 0.25 µL/min. This nLC system was coupled with an Orbitrap Exploris 240 high resolution mass spectrometer equipped with heated electrospray ionization source (Thermo Fisher Scientific, Bremen, Germany). Spray was generated with an integrated column emitter, with tip voltage set at 2.2 kV, capillary voltage at 6 V and capillary temperature at 275 °C. A high-resolution full Fourier-Transform Mass Spectrometry (FTMS) profile spectrum and MS/MS spectra of top 50 ions (scan range 200–3000 m/z, resolving power 60 000, 1 microscan) was acquired using Xcalibur (4.4) software (Thermo Fisher Scientific) with the precursor mass tolerance of 10 ppm. The experiments were done in duplicate. Identification of protein and post-translational modifications (PTMs) was performed using the PEAKS Studio X Pro software program (Bioinformatics Solutions Inc., Waterloo, ON, Canada). Signature MS/MS spectra were searched using PEAKS DB and PEAKS PTM algorithms against a database consisting of a UniProtKB (tremble and reviewed) subfamily Gallus Gallus (taxon identifier 9031, 30 501, downloaded on August 17, 2021 from http://www.uniprot.org/) and cRAP (the common Repository of Adventitious Proteins) database (downloaded on August 17, 2021 from http://www.thegpm.org/crap/). Oxidation (Met) and deamidation (Gln, Asn) were considered as variables, with carbamidomethylation (Cys) set as fixed in the PEAKS DB algorithm. In the PEAKS PTM algorithm, an PTM search was undertaken using a list of 313 PTM items. Up to two missed trypsin cleavages with non-specific cleavages at both ends of a peptide were allowed. Mass tolerances were set to ±10 ppm for parent ions and ± 0.02 Da for fragment ions. Protein filters were as follows: protein −10 lgP ≥ 20, proteins unique peptides ≥ 1, and "A" Score for confident PTMs identification of at least 50. Peptide filters were as follows: false discovery rate for peptide-spectrum matches < 0.5%; therefore, the resulting false discovery rate of the peptide sequence was lower than 1% (for more details check Smiljanic et al, 2019)[33].

When identifying peptides with the PEAKS Studio X pro software package, all trypsin-derived peptides were taken into account, together with peptides generated by hydrolysis catalyzed by an enzyme of unknown specificity, to

obtain all semi-tryptic and non-tryptic peptides. All semi-tryptic or non-tryptic peptides were extracted and overlapped with the peptides found in the control (**Supplementary Table 1**) in order to find peptides generated only by the action of Cu^{II}WD/Asc. The sample preparation steps involved in peptide mapping are also sources of non-enzymatic PTMs. Alkaline pH, used during all steps of sample preparation, induces deamidation and disulfide bond scrambling and oxidation of methionine.

Sample treatment before analysis is necessary and can limit the information obtained. Because of the high tendency of oxidation at Cys residues during processing, reduction and alkylation is carried out. This therefore results in loss of most information about oxidant-mediated changes at Cys residues. Several studies have shown that reduction and alkylation can also decrease the levels of other modifications (e.g. 3-chloro-Tyr). Sequencing without alkylation and reduction can decrease sequence coverage.[34]

2. Supplementary discussion

2.1. Cleavage of HEWL

2.1.1. Oxidative cleavage

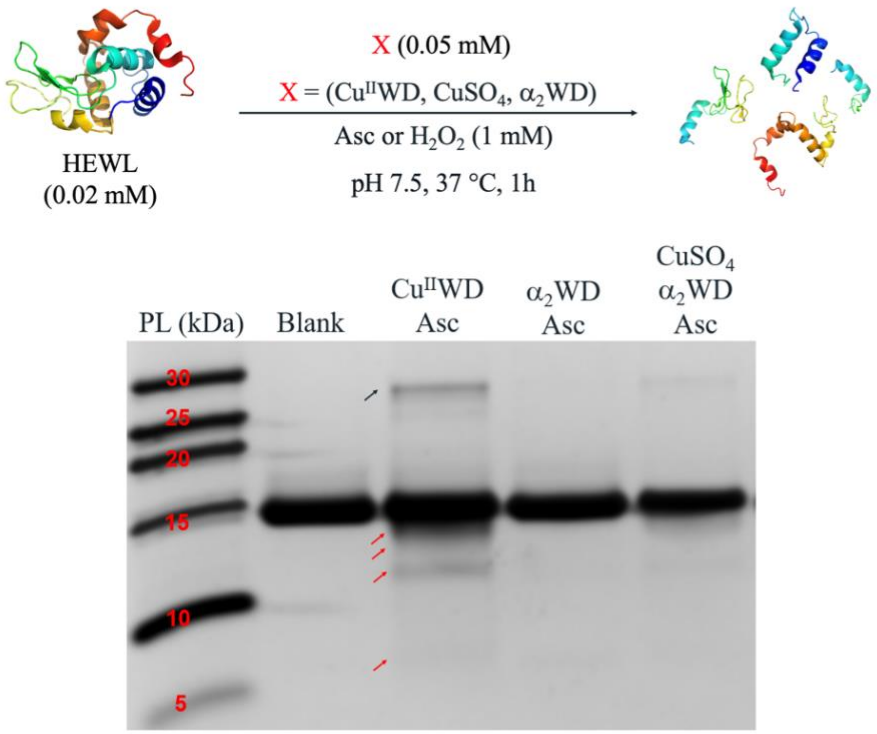

Supplementary Figure 1. HEWL cleavage by different catalysts. Silver stained SDS-PAGE gel of the cleavage of HEWL (0.02 mM) in presence of Cu^{II}WD, α_2WD or $CuSO_4$ (0.05 mM/), Asc (1 mM), at pH 7.4 and 37 °C for 1h. Red arrows indicate the produced protein fragments. The black arrow indicates the bands from HEWL dimerization. Figure shows a representative gel. All experiments were repeated independently three times and provided similar results. Source data are provided as a Source Data file.

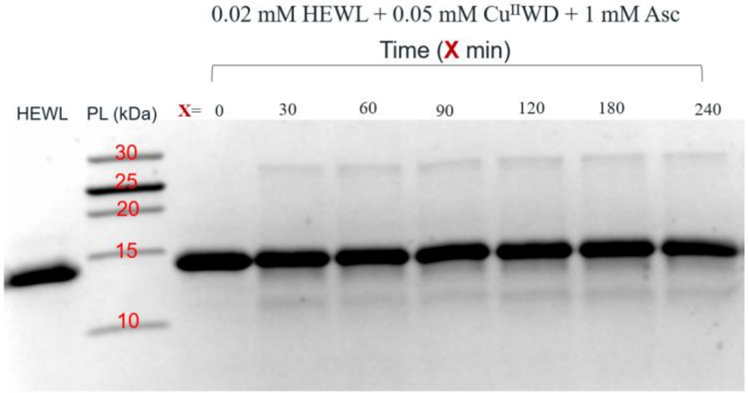

Supplementary Figure 2. HEWL cleavage by Cu^{II}WD/Asc over time. Coomassie stained SDS-PAGE gel of the cleavage of HEWL (0.02 mM) in presence of Cu^{II}WD and Asc at pH 7.5 and 37 °C for 1h. Figure shows a representative gel. All experiments were repeated independently two times and provided similar results. Source data are provided as a Source Data file.

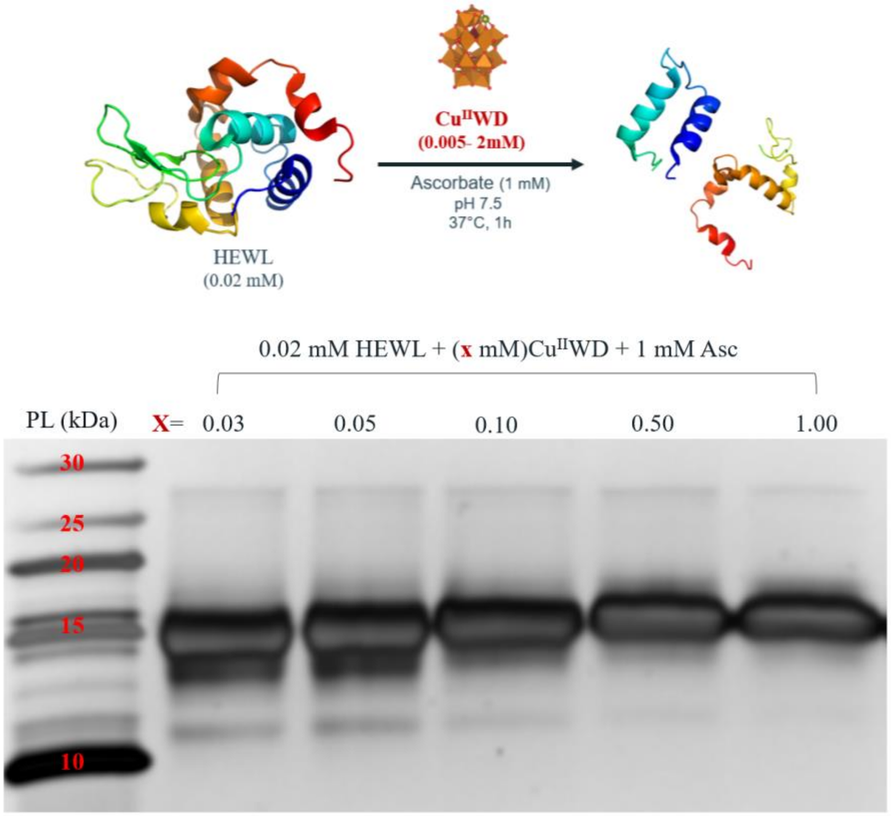

Supplementary Figure 3. HEWL cleavage by different concentrations of Cu^{II}WD in the presence of Asc. Silver stained SDS-PAGE gels of HEWL (0.02mM) cleavage in presence of Asc (1 mM) and different concentration of Cu^{II}WD (0.03-1.00 mM), at pH 7.5 and 37 °C for 1h. Figure shows a representative gel. All experiments were repeated independently two times and provided similar results. Source data are provided as a Source Data file.

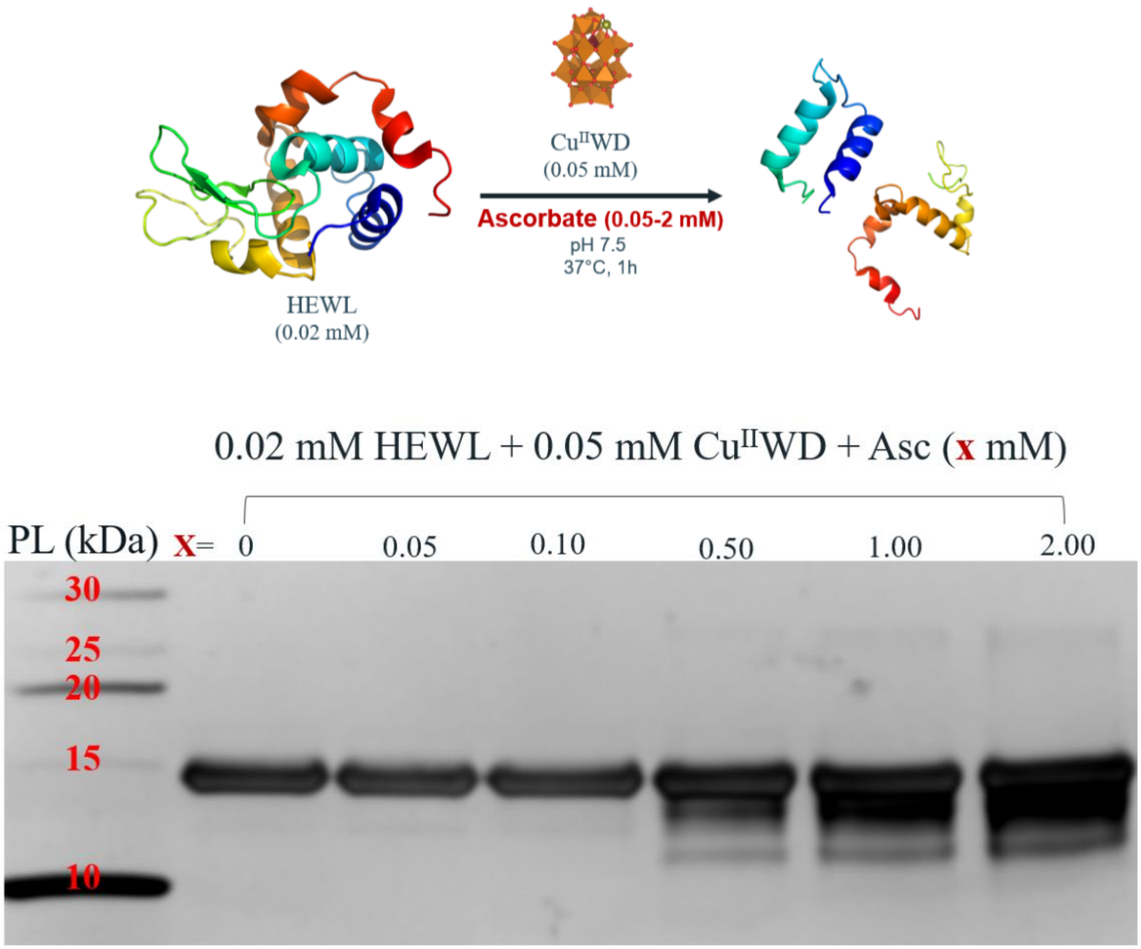

Supplementary Figure 4. HEWL cleavage by Cu^{II}WD and different concentrations of Asc. Silver stained SDS-PAGE gel of HEWL (0.02 mM) cleavage in presence of Cu^{II}WD (0.05 mM) and different concentrations of Asc (0.05-2.00 mM), at pH 7.5 and 37 °C for 1h. Figure shows a representative gel. All experiments were repeated independently two times and provided similar results. Source data are provided as a Source Data file.

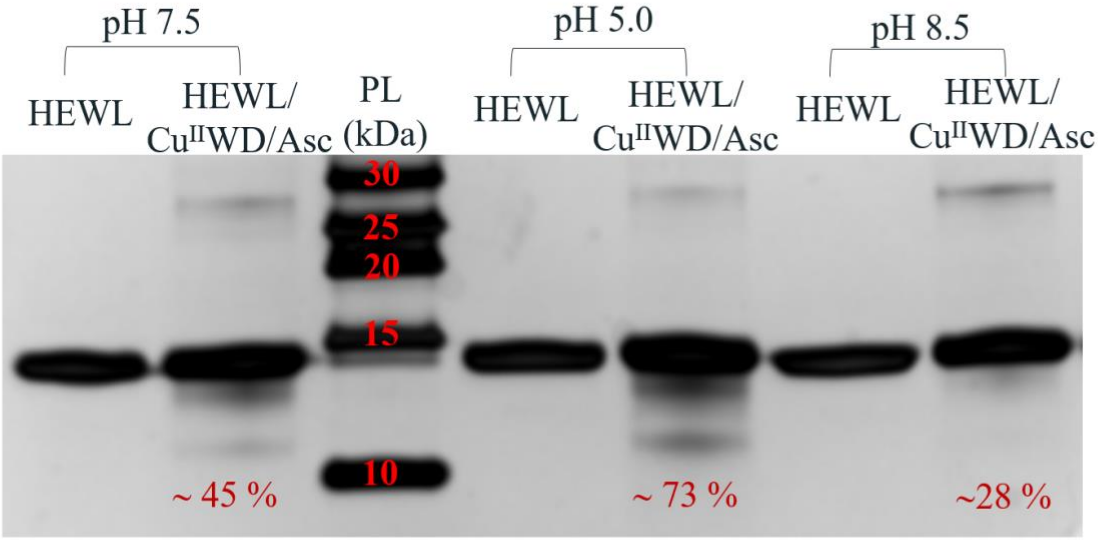

Supplementary Figure 5. HEWL cleavage by Cu^{II}WD/Asc under different pH values. Silver stained SDS-PAGE gel of HEWL (0.02 mM) cleavage in presence of Cu^{II}WD (0.05 mM) and Asc (1mM), at different pH values at 37 °C for 1h. (45, 73 and 28 are the % of HEWL cleaved). Figure show a representative gel. All experiments were repeated independently two times and provided similar results. Source data are provided as a Source Data file.

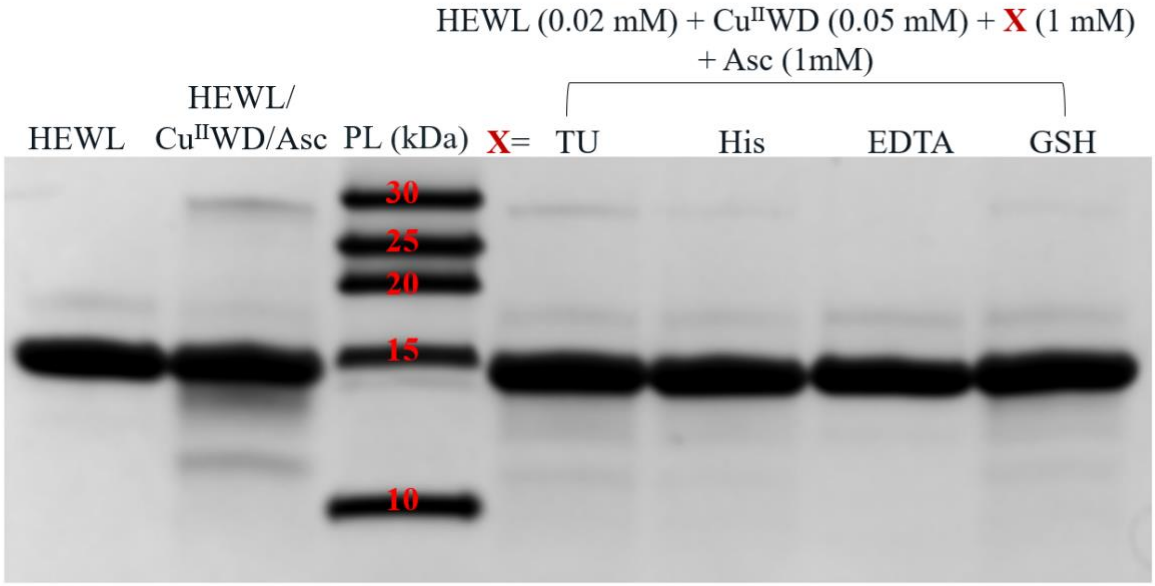

Supplementary Figure 6. HEWL cleavage by Cu^{II}WD/Asc in the presence of different inhibitors. Silver stained SDS-PAGE gel of HEWL (0.02 mM) cleavage in presence of Cu^{II}WD (0.05 mM), Asc (1.00 mM) and inhibitors (1.00 mM), at pH 7.5 and 37 °C for 1h. Figure shows a representative gel. All experiments were repeated independently two times and provided similar results. Source data are provided as a Source Data file.

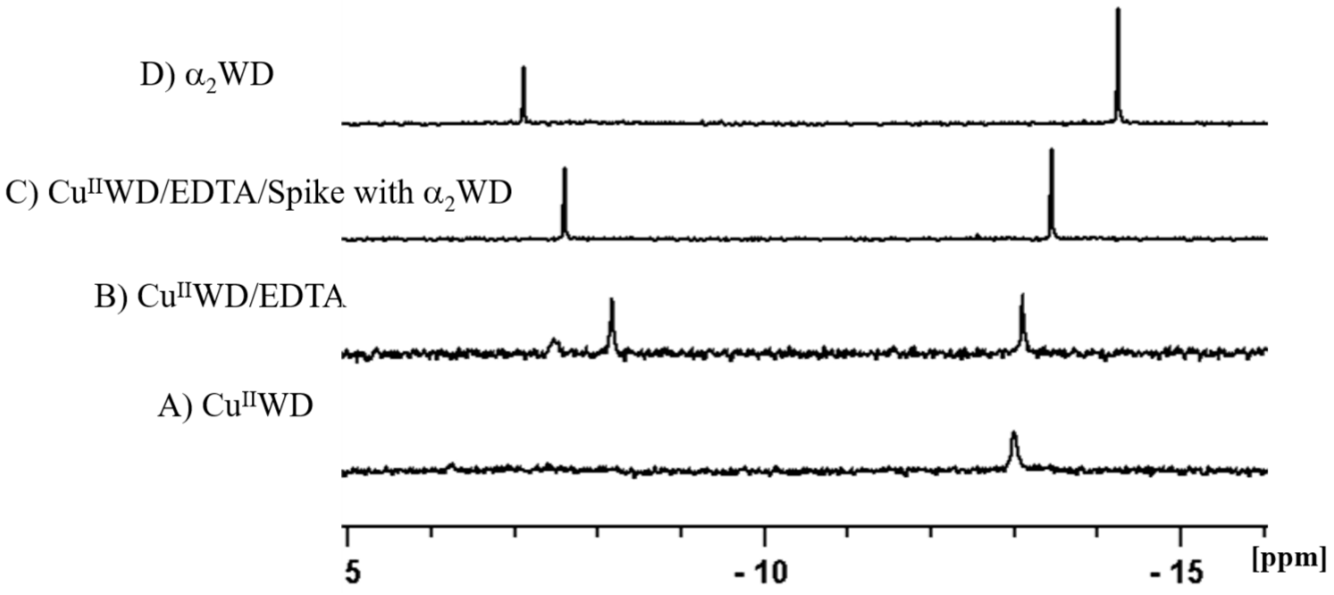

Supplementary Figure 7. ^{31}P NMR spectra of Cu^{II}WD in the absence and presence of EDTA. (A) 2 mM of Cu^{II}WD; (B) 2 mM Cu^{II}WD in presence of 2 mM EDTA; and (C) 2 mM Cu^{II}WD/ 2 mM EDTA spiked with α_2WD; (D) 2 mM α_2WD . At pH 7.5 and 37 °C for 1h.

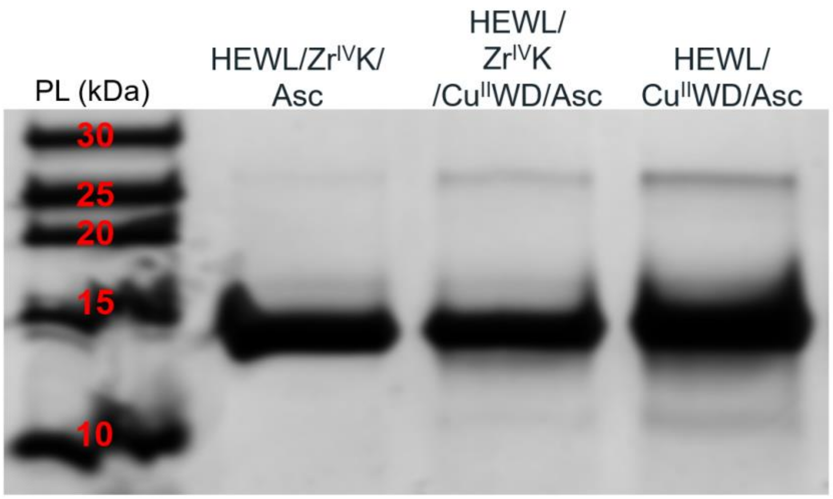

Supplementary Figure 8. HEWL cleavage by Cu^{II}WD/Asc in the presence of Zr^{IV}K. Silver stained SDS-PAGE gel of HEWL (0.02 mM) cleavage in presence of Cu^{II}WD or Zr^{IV}K (0.05 mM), Asc (1.00 mM), at pH 7.5 and 37 °C for 1h. Figure show a representative gel. All experiments were repeated independently two times and provided similar results. Source data are provided as a Source Data file.

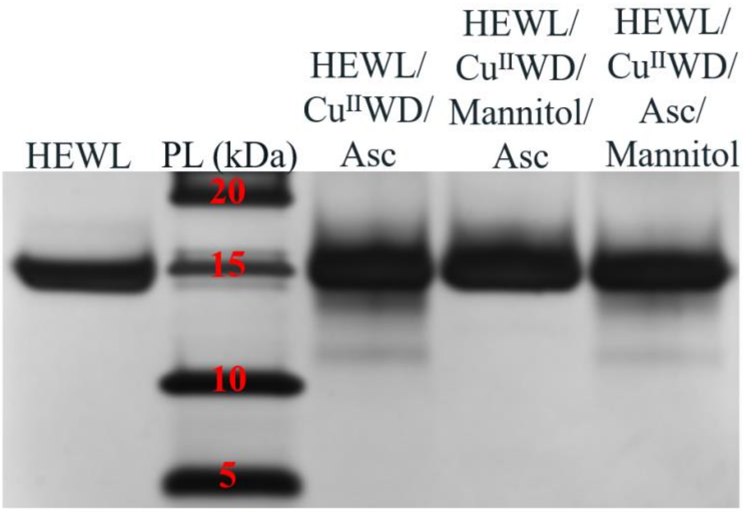

Supplementary Figure 9. **HEWL cleavage by Cu^{II}WD/Asc in the presence of Mannitol.** Silver stained SDS-PAGE gel of HEWL (0.02 mM) cleavage in presence of Cu^{II}WD, Asc (1.00 mM), and mannitol (1.00 mM), at pH 7.5 and 37 °C for 1h. Figure show a representative gel. All experiments were repeated independently two times and provided similar results. Source data are provided as a Source Data file.

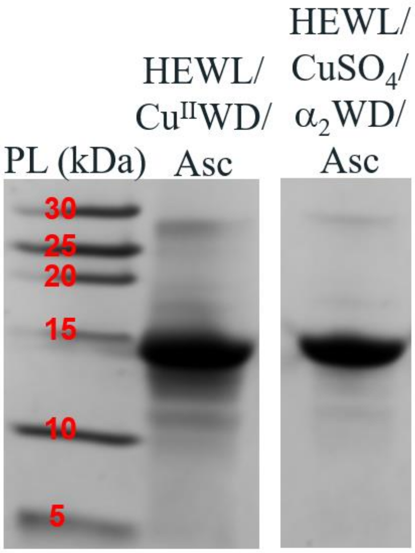

Supplementary Figure 10. Comparison of HEWL cleavage by different Cu catalysts. Silver stained SDS-PAGE gel of HEWL (0.02 mM) cleavage in presence of 0.05 mM Cu^{II}WD or $CuSO_4$/α_2WD (0.05 mM), Asc (1.00 mM) at pH 7.4 and 37 °C for 1h. Figure show representative gels. All experiments were repeated independently three times and provided similar results. Source data are provided as a Source Data file.

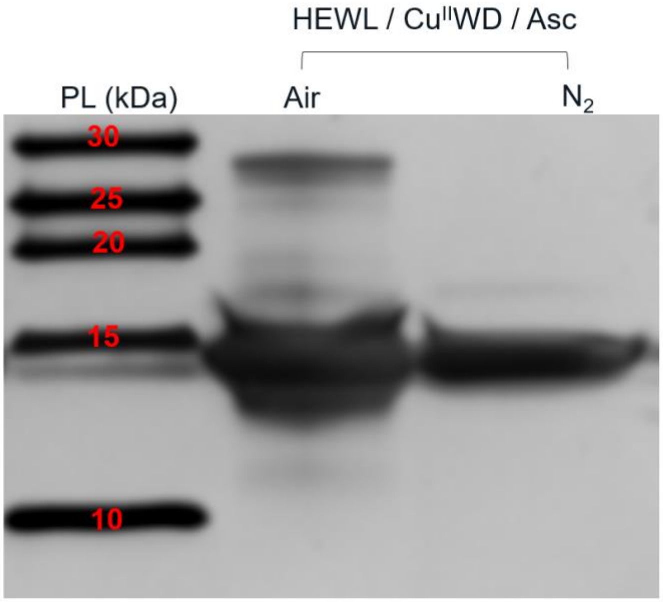

Supplementary Figure 11. HEWL cleavage by Cu^{II}WD/Asc under N_2 atmosphere. Silver stained SDS-PAGE gel of HEWL (0.02 mM) cleavage in presence of Cu^{II}WD (0.05 mM), Asc (1.00 mM), at pH 7.5 and 37 °C for 1h under air or N_2. Figure show a representative gel. All experiments were repeated independently two times and provided similar results. Source data are provided as a Source Data file.

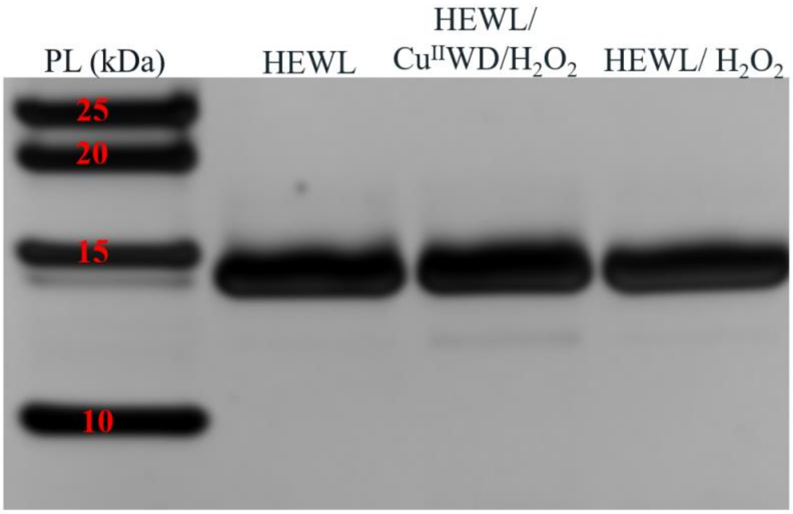

Supplementary Figure 12. HEWL cleavage by Cu^{II}WD/H_2O_2. Silver stained SDS-PAGE gel of HEWL (0.02 mM) cleavage in presence of Cu^{II}WD (0.05 mM) and/or H_2O_2 (1.00 mM), at pH 7.5 and 37 °C for 1h. Figure shows a representative gel. All experiments were repeated independently two times and provided similar results. Source data are provided as a Source Data file.

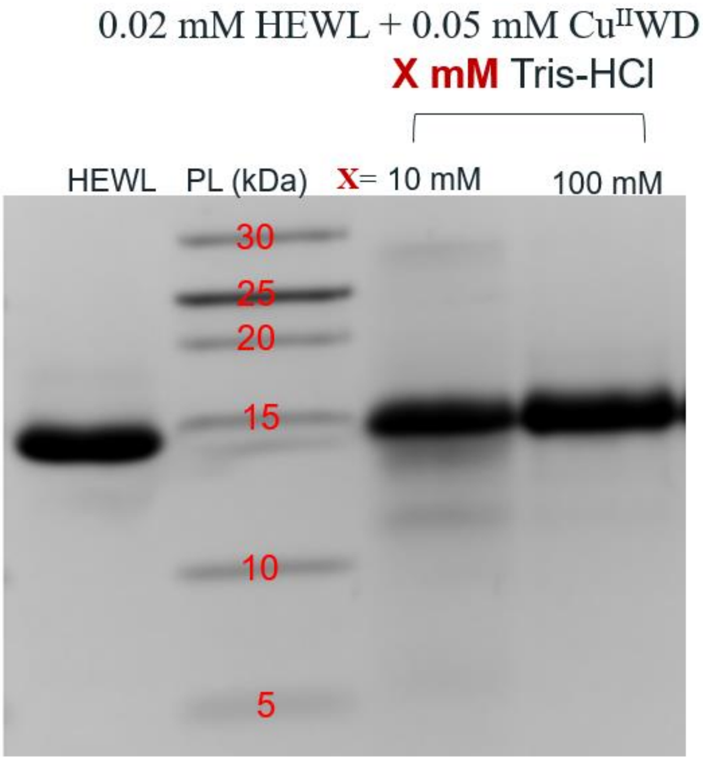

Supplementary Figure 13. HEWL cleavage by Cu^{II}WD/Asc under different buffer concentration. Coomassie stained SDS-PAGE gel of the cleavage of HEWL (0.02 mM) in presence of Cu^{II}WD and Asc at different buffer concentration, pH 7.4 and 37 °C for 1h. Figure shows a representative gel. All experiments were repeated independently two times and provided similar results. Source data are provided as a Source Data file.

2.1.2. Hydrolytic cleavage

The cleavage of HEWL via Cu^{II}WD, in the absence of Asc, was monitored at pH 7.5, 60 °C for 3 days. **Figure S14** shows that in presence of Cu^{II}WD only three bands were produced with (11.5 kDa, 9.1 kDa and 7.6 kDa).

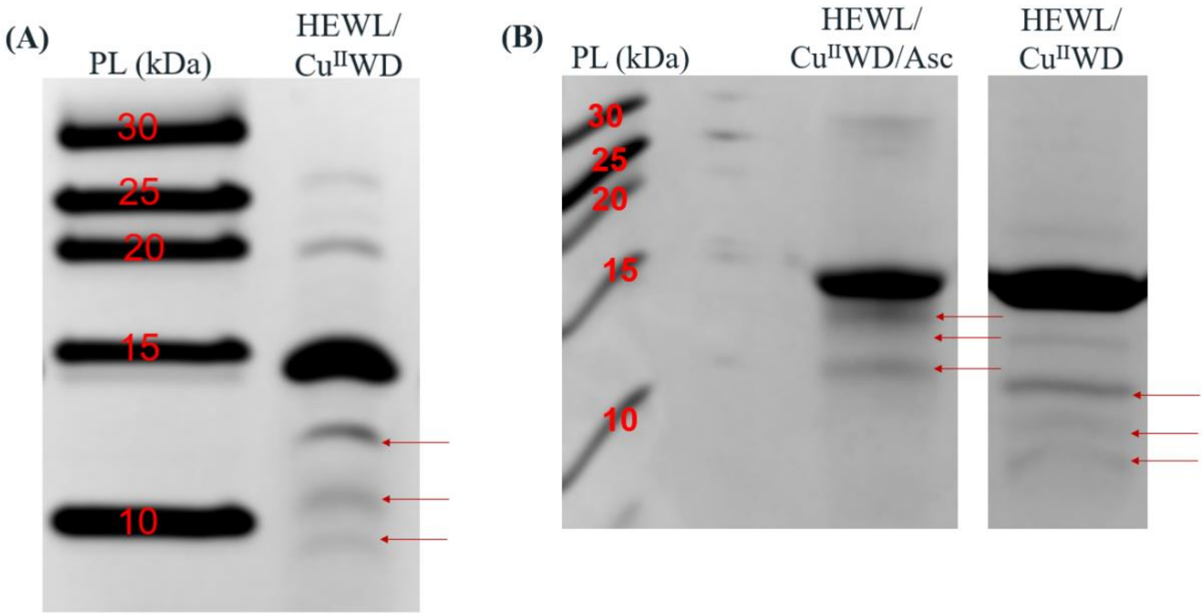

Supplementary Figure 14. **HEWL cleavage by Cu^{II}WD.** (A) Silver stained SDS-PAGE gel of HEWL (0.02 mM) incubation in presence Cu^{II}WD (2 mM) at pH 7.5, 60 °C for 3 days. (B) Comparison of the fragments under hydrolytic and oxidative conditions using a single coomassie stained SDS-PAGE gel of HEWL in presence Cu^{II}WD (0.05 mM)/Asc (1 mM) or 2 mM Cu^{II}WD at pH 7.5, 60 °C for 3 days pH 7.4 and 37 °C for 1h. Figures show representative gels. All experiments were repeated independently three times and provided similar results. Source data are provided as a Source Data file.

Supplementary Figure 14, unambiguously, reveals that the bands produced from the cleavage of HEWL via Cu^{II}WD is different from Cu^{II}WD /Asc. This could be attributed to the difference in cleavage pathway since Cu^{II}WD is most plausibly cleave protein through a hydrolytic pathway. **Supplementary Fig. 15** shows two proposed pathways of the hydrolytic cleavage of the peptide bond based on literature.[2] In the first pathway (**Supplementary Fig. 15-A**) the metal could activate a coordinated hydroxyl or water which eventually will attack the amide carbonyl group. In the second one (**Supplementary Fig. 15-B**), the metal act as Lewis acid by activating an amide carbonyl towards nucleophilic attack by hydroxyl or water molecule from the solvent. Both mechanisms require at least one free coordination site at the catalyst in order to hydrolyze the peptide bond.

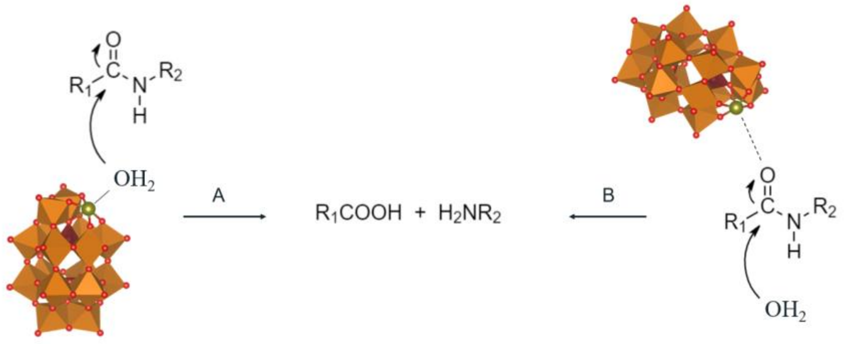

Supplementary Figure 15. Proposed mechanisms of the hydrolytic cleavage of the peptide bond.

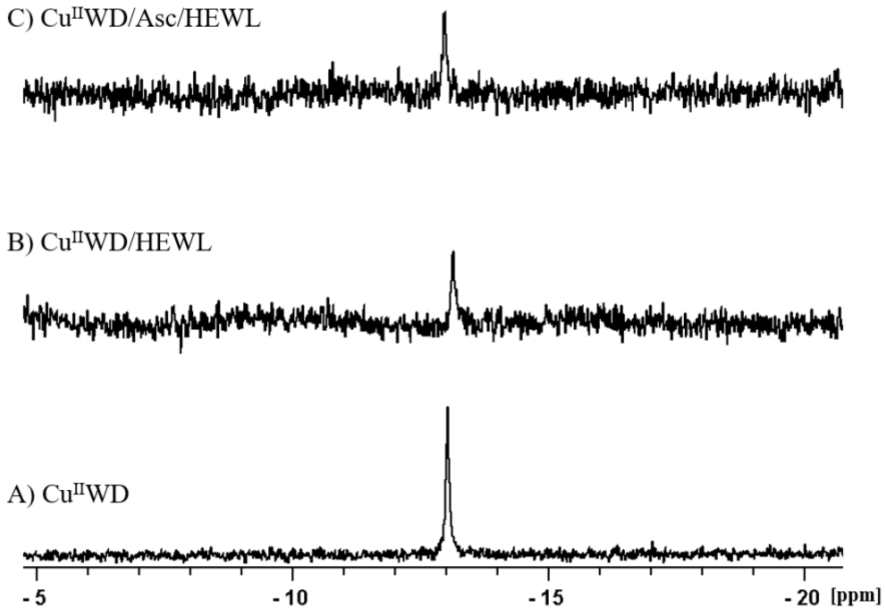

Supplementary Figure 16. **^{31}P NMR spectra of Cu^{II}WD**. (A) 2 mM of Cu^{II}WD; (B) 2 mM Cu^{II}WD in presence of 0.02 mM HEWL; and (C) 2 mM Cu^{II}WD in presence of 0.02 mM HEWL and 2 mM Asc. At pH 7.5 and 60 °C for 1 day.

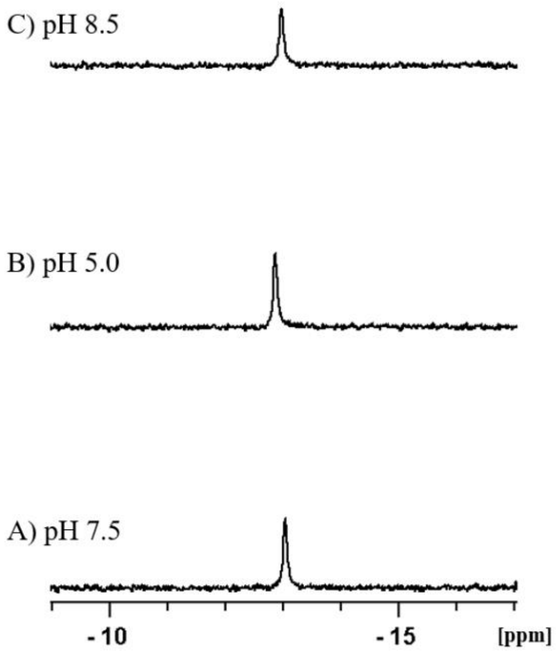

Supplementary Figure 17. ^{31}P NMR spectra of Cu^{II}WD. 5 mM Cu^{II}WD was monitored at different pH values at 60 °C for 1 day.

2.2. UV-Vis spectroscopy

Supplementary Fig. 18 shows no change in the UV-Vis spectrum of Cu^{II}WD before and after the reaction with HEWL/Asc, which indicates the stability of Cu^{II}WD under reaction conditions. The increase in absorption intensity is due to the absorption of Asc (λ_{max}= 265 nm)[3] and HEWL (λ_{max} = 220 and 280 nm). [4]

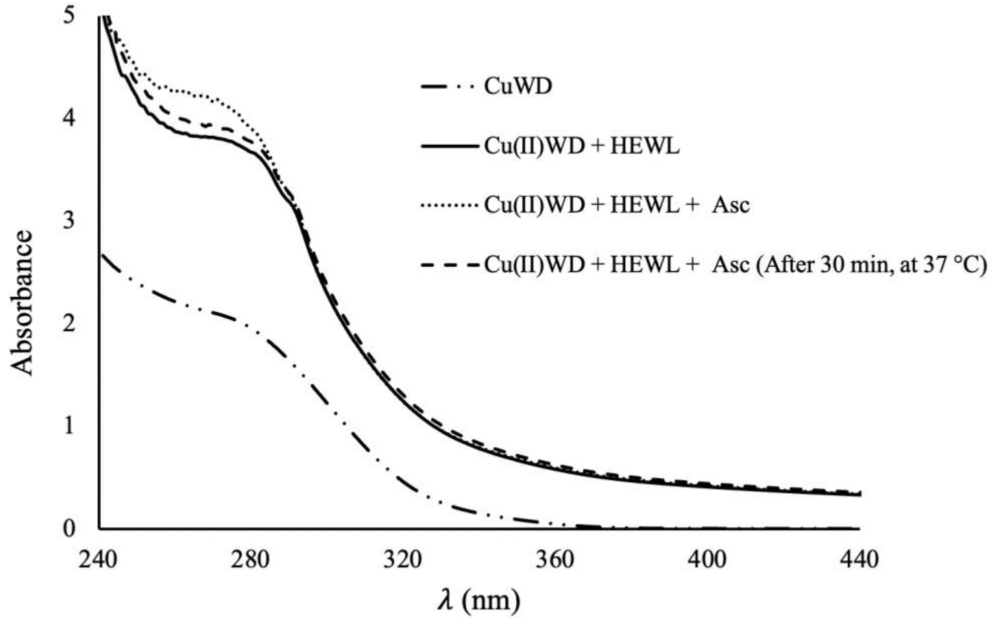

Supplementary Figure 18. UV-Vis absorbance spectra of Cu^{II}WD. Cu^{II}WD (0.05 mM) was measured in presence and absence of HEWL (0.02 mM) and/or Asc (1 mM) at pH 7.5. UV-Vis measurement was repeated two times with similar results on the same sample (technical replicates). Source data are provided as a Source Data file.

2.3.HEWL and Cu^{II}WD interaction studies:

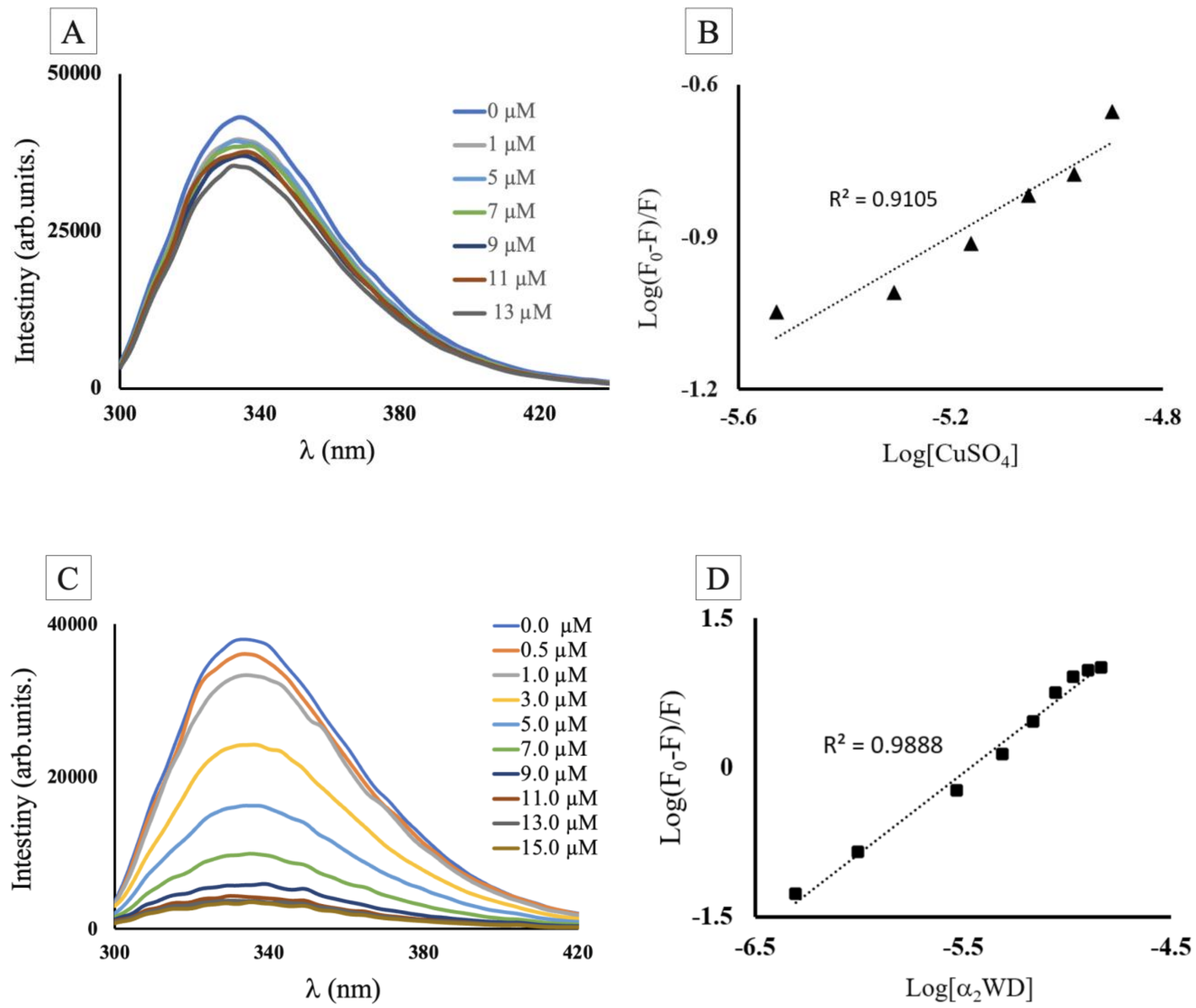

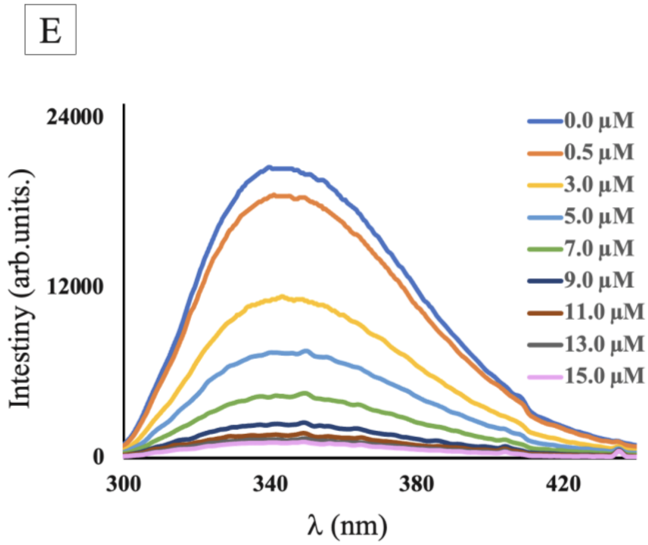

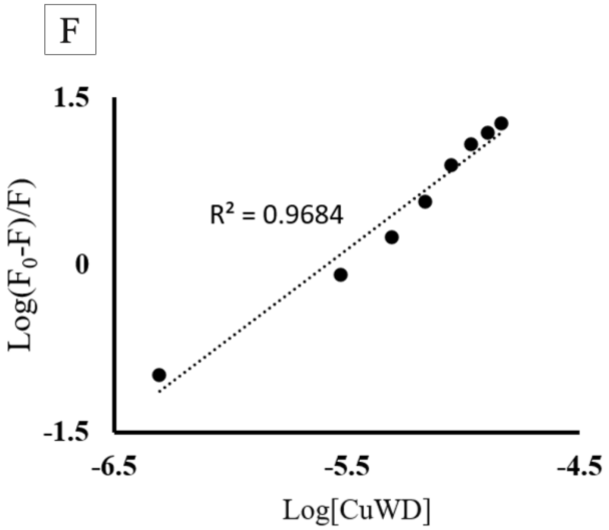

Supplementary Figure 19. Trp fluorescence quenching spectra of HEWL in presence of different quenchers. HEWL (10.0 µM) in the presence of: (A) $CuSO_4$ (0 – 13 µM); (C) α_2WD (0 – 15 µM); (E) Cu^{II}WD (0 – 15 µM) at 25 °C and pH 7.5, and the corresponding plots of the derived Stern–Volmer plot: (B) $CuSO_4$; (D) α_2WD; (F) Cu^{II}WD. Where (F) is maximum fluorescence intensity of protein only and (F_0) is maximum fluorescence intensity of protein in presence of the quencher. measurement was repeated three times with similar results on the same sample (technical replicates). Source data are provided as a Source Data file.

2.3.1. ^{1}H NMR spectroscopy

Supplementary Fig. 20 shows folded HEWL features spread between 6 and 10 ppm, in addition to peaks < 0 ppm. As a control, ^{1}H NMR spectrum of HEWL in the presence of DTT and SDS was also recorded. DTT and SDS are known to cause effective protein denaturation, which leads to unfolding of the protein. From the data shown in **Supplementary Fig. 20**, most of the protein remains in its native form upon addition of Cu^{II}WD, Asc or both.

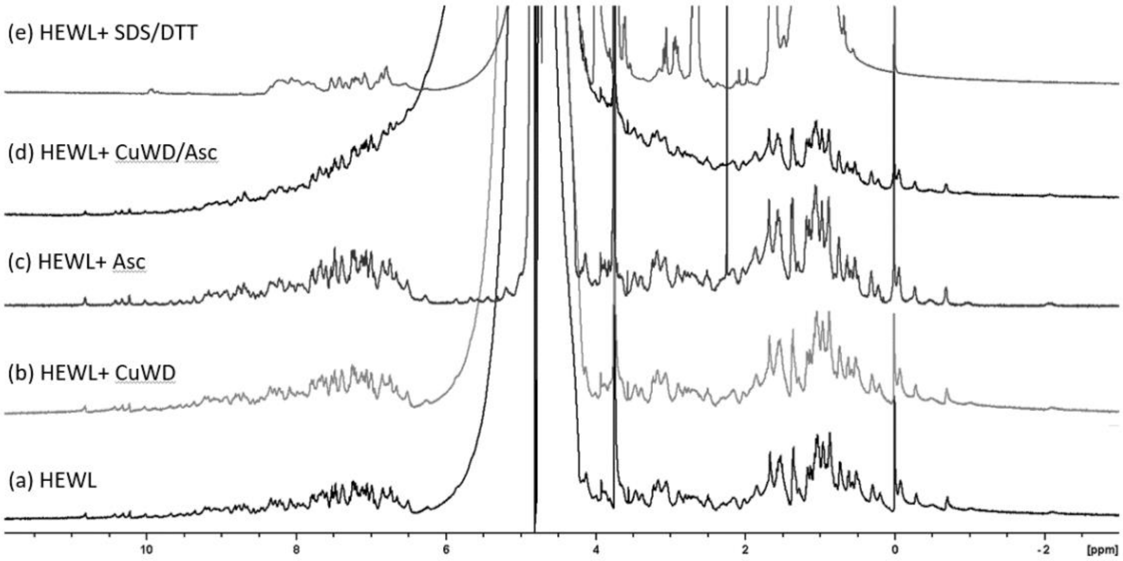

Supplementary Figure 20. ^{1}H NMR spectra of HEWL solutions. a) HEWL (1 mM); b) Cu^{II}WD (0.05 mM); Asc (1 mM); c) Cu^{II}WD /Asc (0.05 mM/1 mM); and e) 10% SDS/DTT (100 mM), at 25 °C and pH 7.5,

2.3.2. Circular dichroism spectroscopy (CD)

The CD spectrum of HEWL in 10 mM tris-HCl (pH 7.4) at room temperature (**Supplementary Fig. 21**) shows a large minimum at λ = 208 nm and a smaller minimum at λ = 222 nm, both characteristic for α-helical structure elements.[5] The minimum at λ = 215 nm, characteristic of β-sheet elements, is less pronounced because HEWL contains only a minor β-strand region.[5,6] **Supplementary Fig. 21** shows that Cu^{II}WD does not change the overall shape of the HEWL CD spectrum but decrease the intensity of the negative signals at 208 ,215 and 222 nm. Upon addition of Asc (1 mM), a drastic change to HEWL CD spectrum was observed which in agreement with literature.[7] The minima at λ = 215 and l=222 nm was almost lost while the peak at λ = 208 nm was less affected. Addition of Cu^{II}WD (0.05 mM) leads to restore minima at λ = 215 and λ =222 nm but decrease their intensity as well as minimum at λ = 208 nm.

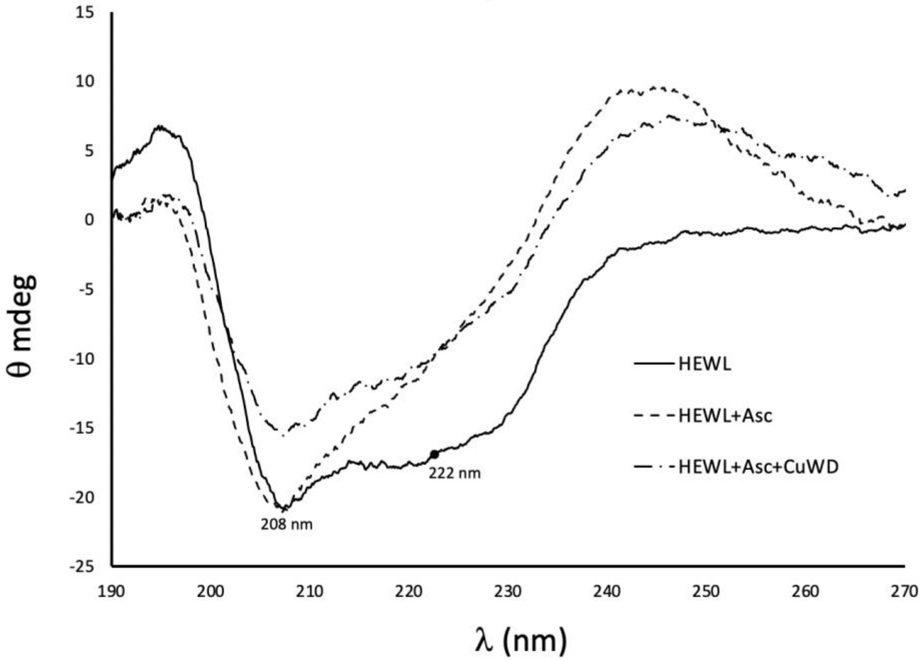

Supplementary Figure 21. **CD spectra of HEWL/Cu^{II}WD/Asc**. CD evidence changes in HEWL (0.01 mM) secondary structure in the presence of Cu^{II}WD (0.05 mM), Asc (1 mM) or both at 10 mM tris-HCl buffer pH 7.5 and 25 °C**.** measurement was repeated two times with similar results on the same sample (technical replicates). Source data are provided as a Source Data file.

2.4. Mechanistic studies

To probe whether the Cu^{II}WD mediated HEWL cleavage in the presence of Asc follows an oxidative pathway, several mechanistic experiments were conducted, based on schemes reported in the literature.[8,9] In the proposed mechanism, Asc reduces Cu^{II}WD to Cu^{I}WD, which in turn is able to reduce an O_2 molecules from the air, leading to the formation of reactive oxygen species (ROS). The ROS presumably induce a radical formation on residues' side chains of HEWL prone to oxidation such as tryptophan, tyrosine, histidine and serine are ultimately cleaving protein through an oxidative pathway.[10,11–13] Therefore, control experiments in the absence of Asc, or in the presence of radical scavengers, were carried. The nature of oxidant was probed in order to investigate the reaction mechanism, as discussed below in detail, and a reaction pathway coherent with the results is presented in (**Supplementary Fig. 24**).

Nature of reaction. The different HEWL fragmentation pattern observed with and without Asc suggests that distinct cleavage reactions take place, supporting that an oxidative cleavage takes place in the presence of Asc. The hydrolysis of HEWL (0.02 mM) in the presence of only Cu^{II}WD (2 mM) at pH 7.4 and 60 °C was much slower than in the presence of Asc, taking 3 days to produce bands with reasonable intensity in the SDS-PAGE analysis.[14,15] Together, these result point to Cu^{II}WD most plausibly cleaving the protein through a hydrolytic pathway in the absence of Asc.[2] In addition, they suggest that reaction undergoes a different pathway in the presence of Asc, most likely an oxidative one.[16–18,19,20,21]

Radical scavengers. Overall conversion of the protein decreases in the presence of classical radical scavengers or under inert atmosphere, supporting the involvement of radical species in the reaction (**Table 2**). Addition of mannitol (1 mM), a hydroxyl radical scavenger,[22] shortly after mixing Cu^{II}WD /Asc with HEWL, led to a 47% decrease in the protein cleavage, while mixing mannitol with Cu^{II}WD and HEWL before the addition of Asc completely inhibited the cleavage reaction. Further, the readily oxidizable tripeptide glutathione (GSH)[23] had no effect on the reaction when present in low concentration (0.02 mM), but a 78% inhibition was observed when an equimolar amount (1 mM) relative to Asc was used. ^{1}H NMR spectroscopy, **Supplementary Fig. 22**, showed the formation of GSSG, the oxidized form of GSH, which evidence the radical scavenging activity of GSH. Thiourea (1 mM) had a similar effect, inhibiting protein cleavage by 78%.[24]

Procedure for GSH oxidation. A 1.5 mL centrifuge tube was charged with 5 µL of 10 mM Cu^{II}WD stock solution, 5 µL of 200 mM Asc stock solution, 5 µL of 200 mM GSH stock solution and 985 µL of D_2O (final concentration of Cu^{II}WD is 0.05 mM, Asc and GSH both were 1.00 mM). The reaction mixture was homogenized using a vortex. Next, 0.50 mM TMSP-d_4 was added as an internal standard for ^{1}H NMR. Then, 500 µL of the reaction mixture was transferred to an NMR tube.

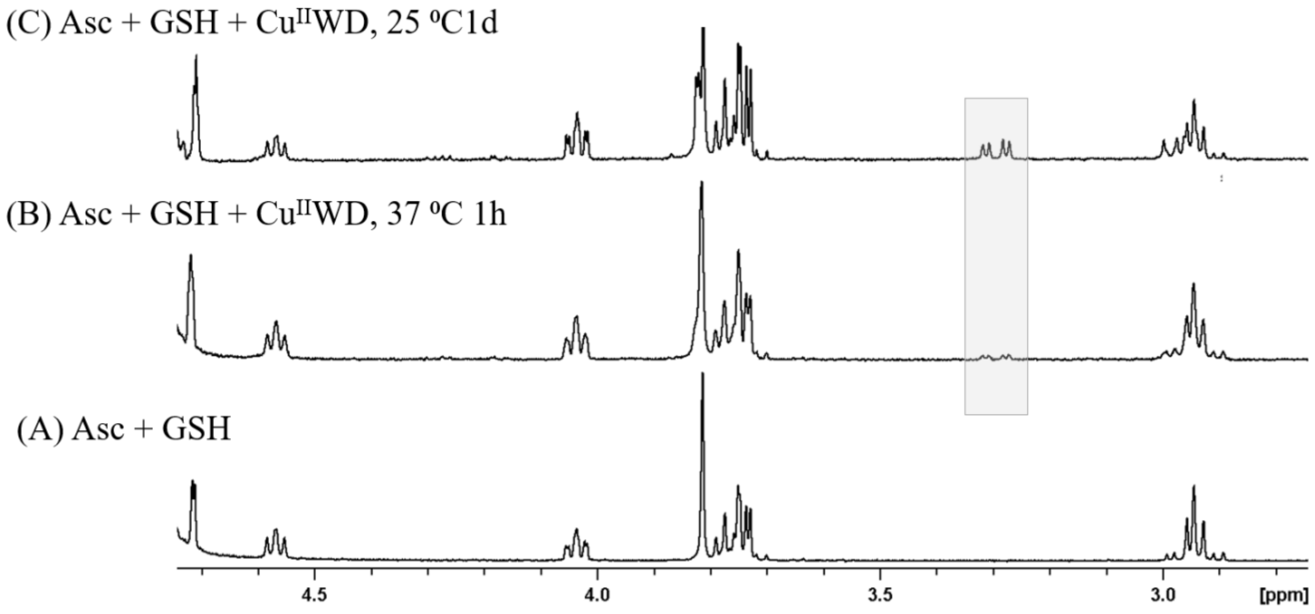

Supplementary Figure 22. ^{1}H NMR spectra of GSH oxidation by Cu^{II}WD/Asc. (A) 2 mM Asc and GSH oxidation in absence of Cu^{II}WD (0.05 mM); (B) 2 mM Asc and GSH oxidation in absence of Cu^{II}WD (0.05 mM).The grey box marked the most indicative peaks which are related to the oxidized GSH (GSSG)

Role of ascorbate. The *in situ* formation of an ascorbyl radical was confirmed by electron paramagnetic resonance spectroscopy (EPR). The EPR spectra of Cu^{II}WD solutions with and without HEWL were measured under the same conditions under which cleavage reactions were conducted and showed that an ascorbyl radical (Asc• –) was formed immediately after the addition of Asc in both cases (**Figure 3**). Accordingly, the formation of dehydroascorbic acid (DA) upon addition of Cu^{II}WD to an Asc solution was also observed by ^{1}H NMR spectroscopy (**Supplementary Fig. 23**), confirming the stepwise reduction of Cu^{II}WD by Asc in solution. These results strongly support the role of Asc as the sacrificial reductant in the reaction.

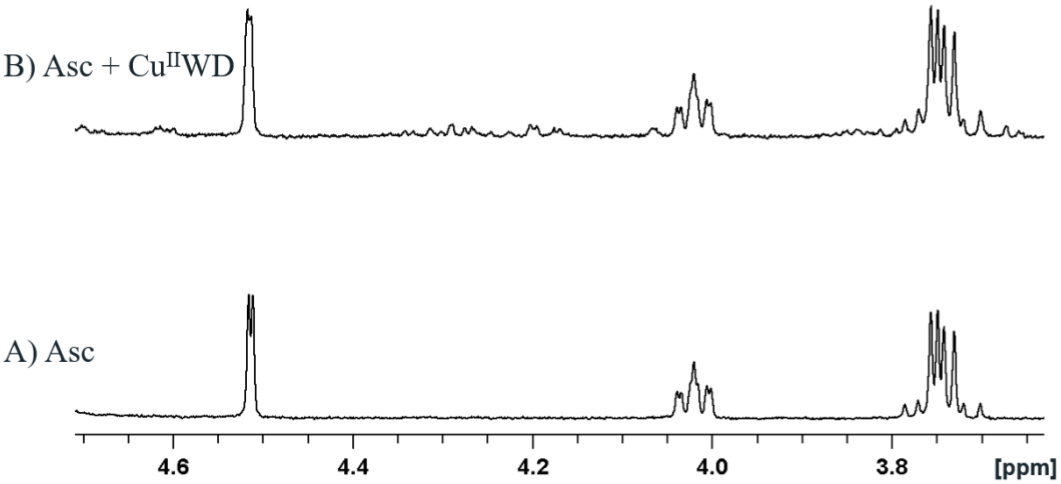

Supplementary Figure 23. ^{1}H NMR spectra of Asc oxidation by Cu^{II}WD. (A) Asc (1.00 mM); (B) Asc (1.00 mM) in the presence of Cu^{II}WD (0.05 mM) where the new peaks indicate the oxidation of Asc at 37 ºC for 30 min.

Nature of oxidant. No HEWL cleavage was observed when the reaction was conducted under an atmosphere of nitrogen (**Supplementary Fig. 11**), corroborating the oxidative character of the reaction, and pointing out the crucial role of oxygen in the mechanism. In general, the reduction of oxygen has been proposed to afford reactive oxygen species like $O_2^{\bullet-}$, O_2^{2-} or $HO^{\bullet}$, which can be protonated in solution to generate peroxides that could further react. However, no cleavage was detected when HEWL was treated with Cu^{II}WD /H_2O_2 (0.05 mM/1 mM) instead of the usual Cu^{II}WD/Asc combination (**Supplementary Fig. 12**), strongly suggesting that any H_2O_2 eventually formed in solution is not responsible for the cleavage observed. This contradicts common mechanistic proposals stating H_2O_2 as an intermediate in similar reactions,[25] though the reactivity of Cu^{II} complexes towards H_2O_2 largely depends on the redox potential of the complex, which may vary widely due to the ligands in the coordination sphere of Cu^{II}, and/or the reaction conditions.[26,27,28] Together, these results suggest a mild oxidant species formed through the reaction of Cu^{I}WD and O_2 might be enabling HEWL oxidative cleavage.[29,30] Importantly, this would also account for the fact that the reaction is not inhibited Asc's known radical scavenger ability,[31] as the oxidant would be produced in low concentration at the vicinity of the protein, unaffected by an excess of Asc.

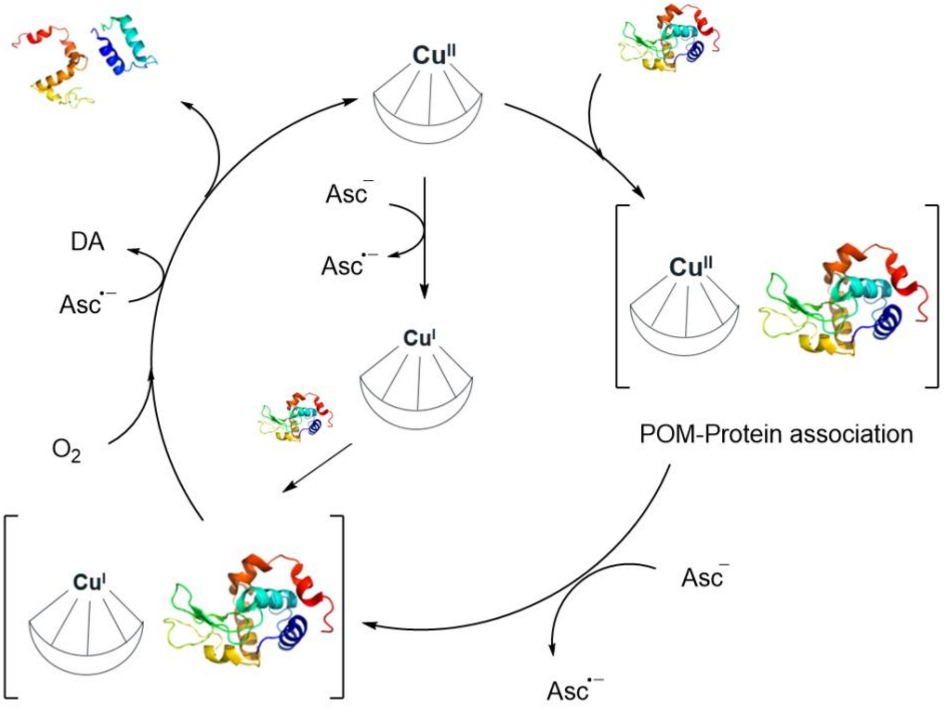

Supplementary Figure 24. The proposed mechanism of the reaction of Cu^{II}WD with Asc

2.5. Identification of oxidative modifications and cleavage sites by nLC-MS/MS.

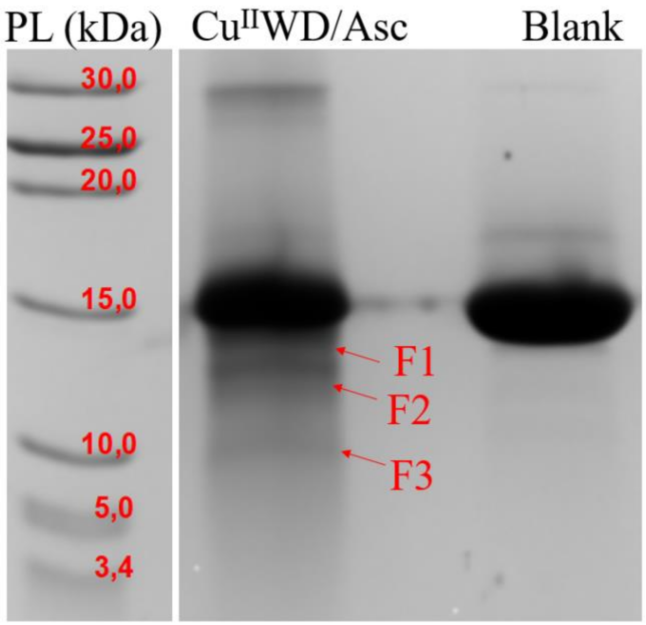

Supplementary Figure 25. HEWL cleavage by Cu^{II}WD/Asc. CBB SDS-PAGE gel of HEWL (0.04 mM) cleavage in presence of Cu^{II}WD (0.1 mM), Asc (4 mM). F1, 2 and 3 are the oxidative cleaved fragments. Figure show a representative gel. All experiments were repeated independently three times and provided similar results.

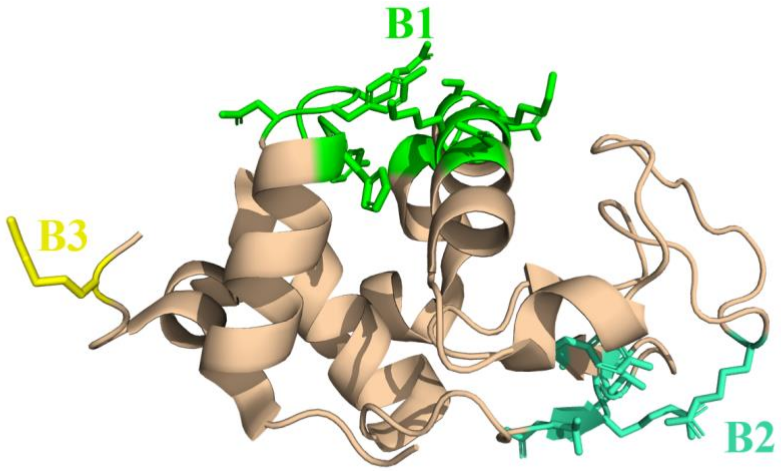

Supplementary Figure 26. Cartoon representation of HEWL structure. Three different binding sites are indicated: B1-[green] (His15, Gly16, Asn19, Tyr20, Arg21, Asn93, Lys96, and Lys97); B2-[greencyan] (Asn44, Arg45, Asn46, Thr47); B3-[yellow] (Arg128).

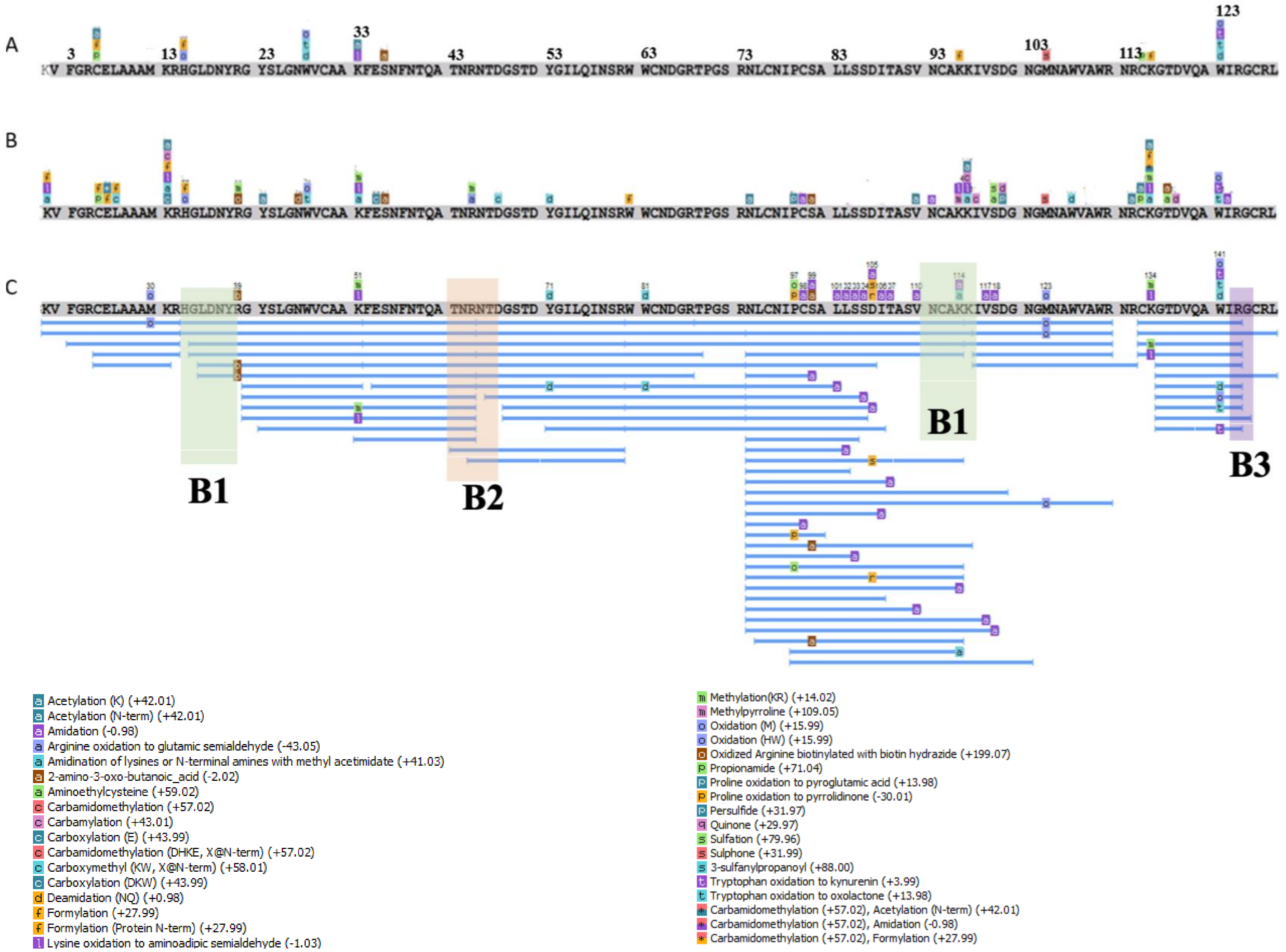
A
3 13 23 33 43 53 63 73 83 93 103 113 123
KV FGRCELAAAM KRHGLDNYRG YSLGNWVCAA KFESNFNTQA TNRNTDGSTD YGILQINSRW WCNDGRTPGS RNLCNIPCSA LLSSDITASV NCAKKIVSDG NGMNAWVAWR NRCKGTDVQA WIRGCRL
B
KV FGRCELAAAM KRHGLDNYRG YSLGNWVCAA KFESNFNTQA TNRNTDGSTD YGILQINSRW WCNDGRTPGS RNLCNIPCSA LLSSDITASV NCAKKIVSDG NGMNAWVAWR NRCKGTDVQA WIRGCRL
C
KV FGRCELAAAM KRHGLDNYRG YSLGNWVCAA KFESNFNTQA TNRNTDGSTD YGILQINSRW WCNDGRTPGS RNLCNIPCSA LLSSDITASV NCAKKIVSDG NGMNAWVAWR NRCKGTDVQA WIRGCRL
B1
B2
B1
B3
Acetylation (K) (+42.01)
Acetylation (N-term) (+42.01)
Amidation (-0.98)
Arginine oxidation to glutamic semialdehyde (-43.05)
Amidination of lysines or N-terminal amines with methyl acetimidate (+41.03)
2-amino-3-oxo-butanoic_acid (-2.02)
Aminoethylcysteine (+59.02)
Carbamidomethylation (+57.02)
Carbamylation (+43.01)
Carboxylation (E) (+43.99)
Carbamidomethylation (DHKE, X@N-term) (+57.02)
Carboxymethyl (KW, X@N-term) (+58.01)
Carboxylation (DKW) (+43.99)
Deamidation (NQ) (+0.98)
Formylation (+27.99)
Formylation (Protein N-term) (+27.99)
Lysine oxidation to aminoadipic semialdehyde (-1.03)
Methylation(KR) (+14.02)
Methylpyrroline (+109.05)
Oxidation (M) (+15.99)
Oxidation (HW) (+15.99)
Oxidized Arginine biotinylated with biotin hydrazide (+199.07)
Propionamide (+71.04)
Proline oxidation to pyroglutamic acid (+13.98)
Proline oxidation to pyrrolidinone (-30.01)
Persulfide (+31.97)
Quinone (+29.97)
Sulfation (+79.96)
Sulphone (+31.99)
3-sulfanylpropanoyl (+88.00)
Tryptophan oxidation to kynurenin (+3.99)
Tryptophan oxidation to oxolactone (+13.98)
Carbamidomethylation (+57.02), Acetylation (N-term) (+42.01)
Carbamidomethylation (+57.02), Amidation (-0.98)
Carbamidomethylation (+57.02), Formylation (+27.99)

Supplementary Figure 27. HEWL amino acid sequence with different modification. (A) Untreated HEWL, (B) Treated intact HEWL, (C) Coverage of HEWL sequence by Cu^{II}WD/Asc generated peptides and the different Cu^{II}WD binding sites are highlighted: Green (B1: His15, Gly16, Asn19, Tyr20, Arg21, Asn93, Lys96, and Lys97); Orange (B2: Asn44, Arg45, Asn46, Thr47); Purple (B3: Arg128). The experiments were done in duplicate.

Supplementary Table 1. All peptides found only in Cu^{II}WD/Asc treated samples (F1, F2, F3). Colors corresponding to regions around binding sites: green-B1, orange- B2, purple- B3.

Entry	Peptide	Start	End	PTM	Sample
1	V.FGRC(+57.02)ELAAAM(+15.99)KR.H	3	14	Carbamidomethylation; Oxidation (M)	F1
2	V.FGRC(+57.02)ELAAAMKR.H	3	14	Carbamidomethylation	F1
3	V.FGRC(+57.02)ELAAAMKR.H	3	14	Carbamidomethylation	F3
4	G.RC(+57.02)ELAAAMKR.H	5	14	Carbamidomethylation	F1, F3
5	R.C(+71.04)ELAAAMKR.H	6	14	Propionamide	F1
6	R.C(+57.02)ELAAAMKR.H	6	14	Carbamidomethylation	F1, F2, F3
7	R.C(+57.02)ELAAAMK(+57.02)R.H	6	14	Carbamidomethylation; Carbamidomethylation (DHKE X@N-term)	F1
8	E.L(+27.99)AAAMKR.H	8	14	Formylation	F1
9	E.L(+58.01)AAAMKR.H	8	14	Carboxymethyl (KW X@N-term)	F1
10	H.GLDNYRGYSLGNWVC(+57.02)AAK.F	16	33	Carbamidomethylation	F1, F2, F3
11	H.GLDNYRGYSLGNWVC(+57.02)AAK.F	16	33	Carbamidomethylation	F1
12	G.LDNYRGYSLGNWVC(+57.02)AAKFESNFNTQATNR.N	17	45	Carbamidomethylation	F1, F2,
13	G.LDNYRGYSLGNWVC(+57.02)AAK.F	17	33	Carbamidomethylation	F1
14	G.LDNYRGYSLGNWVC(+57.02)AAK.F	17	33	Carbamidomethylation	F1
15	G.LDNYRGYSLGNWVC(+57.02)AAK.F	17	33	Carbamidomethylation	F1, F2,
16	G.Y(+79.97)SLGNWVC(+57.02)AAKFESNFNTQATNR.N	23	45	Carbamidomethylation	F1, F2
17	G.Y(+88.00)SLGNWVC(+57.02)AAKFESNFNTQATNR.N	23	45	3-sulfanylpropanoyl; Carbamidomethylation	F1
18	G.Y(+88.00)SLGNWVC(+57.02)AAK.F	23	33	3-sulfanylpropanoyl; Carbamidomethylation	F2
19	Y.SLGNWVC(+57.02)AAK.F	24	33	Carbamidomethylation	F2
20	N.WVC(+57.02)AAKFESNFNTQATNR.N	28	45	Carbamidomethylation	F2, F3
21	N.WVC(+57.02)AAKFESNFNTQATNR.N	28	45	Carbamidomethylation	F2
22	V.CAAKFESNFNTQATNR.N	30	45		F2
23	A.K(+97.02)FESNFNTQATNR.N	33	45	Maleimide	F2
24	A.K(+119.04)FESNFNTQATNR.N	33	45	Pyridylacetyl	F2
25	F.ESNFNTQATNR.N	35	45		F2, F3
26	F.N(+88.00)TQATNRNTDGSTDYGILQINSR.W	39	61	3-sulfanylpropanoyl	F2
27	N.TQATNRN(-.98).T	40	46	Amidation	F2
28	A.TNRNTDGSTDYGILQINSR.W	43	61		F2
29	R.NTDGSTDYGILQIN.S	46	59		F2, F3
30	N.TDGSTDYGILQINSR.W	47	61		F2
31	N.TDGSTDYGILQINSRWWC(+57.02)NDGRTPGSR.N	47	73	Carbamidomethylation	F2
32	N.T(+57.02)DGSTDYGILQINSR.W	47	61	Carbamidomethylation	F2

33	T.D(+58.01)GSTDYGILQINSR.W	48	61	Carboxymethyl (KW X@N-term)	F2
34	D.GSTD(+57.02)YGILQINSR.W	49	61	Carbamidomethylation	F2, F3
35	D.GSTD(+43.99)YGILQINSR.W	49	61	Carboxylation (DKW)	F2
36	R.WWC(+57.02)NDGRTP(-.98).G	62	70	Carbamidomethylation; Amidation	F2
37	R.WWC(+57.02)NDGRTPG.S	62	71	Carbamidomethylation	F2
38	R.NLC(+57.02)NIPC(+57.02)SALLSSDITASVNC(+57 .02)AKKIV(-.98).S	74	99	Carbamidomethylation; Amidation	F2
39	R.NLC(+57.02)NIPC(+57.02)SALLS(-.98).S	74	85	Carbamidomethylation; Amidation	F2, F3
40	R.NLC(+57.02)NIPC(+57.02)S(-.98).A	74	81	Carbamidomethylation; Amidation	F2
41	R.NLC(+57.02)NIPC(+57.02)(-.98).S	74	80	Carbamidomethylation; Amidation	F2
42	R.NLC(+57.02)NIPC(+57.02)SALLSSDITASV(-.98).N	74	92	Carbamidomethylation; Amidation	F2
43	R.NLC(+57.02)NIPC(+57.02)SALLSS(-.98).D	74	86	Carbamidomethylation; Amidation	F2
44	R.NLC(+57.02)NIPC(+57.02)SALL(-.98).S	74	84	Carbamidomethylation; Amidation	F2, F3
45	R.NLC(+57.02)NIPC(+57.02)SALLSSDI(-.98).T	74	88	Carbamidomethylation; Amidation	F2
46	R.NLC(+57.02)NIPC(+57.02)SALLSSD(-.98).I	74	87	Carbamidomethylation; Amidation	F2
47	R.NLC(+57.02)NIPC(+57.02)SALLSSDI.T	74	88	Carbamidomethylation	F2
48	R.NLC(+57.02)NIPC(+57.02)SALLSS.D	74	86	Carbamidomethylation	F2
49	R.NLC(+57.02)NIPC(+57.02)SALLSSDI.T	74	88	Carbamidomethylation	F2, F3
50	R.NLC(+57.02)NIPC(+57.02)SALL.S	74	84	Carbamidomethylation	F2, F3
51	R.NLC(+57.02)NIPC(+57.02)SAL(-.98).L	74	83	Carbamidomethylation; Amidation	F2
52	R.NLC(+57.02)NIPC(+57.02)SALLSSDITASVNC(+57 .02)AKKIVS(-.98).D	74	100	Carbamidomethylation; Amidation	F2
53	R.NLC(+57.02)NIP(-30.01)C(+57.02)SA.L	74	82	Carbamidomethylation; Proline oxidation to pyrrolidinone	F2, F3
54	R.NLC(+57.02)N(+.98)IPC(+57.02)SALLSSDITASVN C(+57.02)AKKIVSD.G	74	101	Carbamidomethylation, Deamidation	F2
55	R.NLC(+57.02)NIPC(+57.02)SALLS.S	74	85	Carbamidomethylation	F2
56	R.NLC(+57.02)NIPC(+57.02)SALLSSDIT(-.98).A	74	89	Carbamidomethylation; Amidation	F2
57	R.NLC(+57.02)NIPC(+57.02)SALLSSDITASVNC(+57 .02)AKK(+57.02).I	74	97	Carbamidomethylation	F2
58	R.NLC(+57.02)NIPC(+57.02)SALLSSDITAS(-.98).V	74	91	Carbamidomethylation; Amidation	F2
59	R.NLC(+57.02)NIPC(+57.02)S(-18.01)ALL.S	74	84	Carbamidomethylation; Dehydration	F2
60	N.LC(+57.02)NIPC(+57.02)SALLSSD(+57.02)ITASV NC(+57.02)AK.K	75	96	Carbamidomethylation	F2
61	N.LC(+57.02)NIPC(+57.02)SALLSSDITASVNC(+57. 02)AK.K	75	96	Carbamidomethylation	F2
62	N.LC(+57.02)NIPC(+57.02)S(+126.10)ALLSSDITASV NC(+57.02)AK.K	75	96	Carbamidomethylation	F2
63	N.LC(+57.02)NIPC(+57.02)S(+136.00)ALLSSDITASV NC(+57.02)AKK.I	75	97	Carbamidomethylation	F2
64	N.LC(+57.02)NIPC(+57.02)SALLS(+154.00)SDITASV NC(+57.02)AK.K	75	96	Carbamidomethylation	F2
65	N.LC(+57.02)NIPC(+57.02)S(+121.04)ALLSSDITASV NC(+57.02)AK.K	75	96	Carbamidomethylation	F2

66	N.LC(+57.02)NIPC(+57.02)S(+79.97)ALLSSD(+57.02)ITASVNC(+57.02)AK.K	75	96	Carbamidomethylation	F2
67	N.LC(+57.02)NIPC(+57.02)S(+59.02)ALLSSDITASVNC(+57.02)AKK.I	75	97	Carbamidomethylation	F2
68	L.C(+57.02)NIPC(+57.02)S(+380.15)ALLSSDITASVNC(+57.02)AKK.I	76	97	Carbamidomethylation	F2
69	I.P(+13.98)C(+57.02)SALLSSDITASVNC(+57.02)AK.K	79	96	Proline oxidation to pyroglutamic acid; Carbamidomethylation	F2
70	I.PC(+57.02)SALLSSDITASVNC(+57.02)AKK(+31.99)IVSDGN.G	79	103	Carbamidomethylation	F2
71	I.PC(+57.02)SALLSSDITASVNC(+57.02)AKK(+14.02)IVS(+79.97).D	79	100	Carbamidomethylation	F2
72	I.PC(+57.02)SALLSSDITASVNC(+57.02)AK(+41.03).K	79	96	Carbamidomethylation; Amidination of lysines or N-terminal amines with methyl acetimidate	F2
73	I.PC(+57.02)SALLSSDITAS(-20.03)VNC(+57.02)AKKIVSDGNG.M	79	104	Carbamidomethylation	F2
74	I.PC(+57.02)SALLSSDITASVNC(+57.02)AK(+27.99)KIVSDGN.G	79	103	Carbamidomethylation	F2
75	I.PC(+57.02)SALLSSDITASVNC(+57.02)AKKIVS.D	79	100	Carbamidomethylation	F2
76	C.SALLSSD(+61.92)ITASVNC(+57.02)AK.K	81	96	Replacement of proton by copper; Carbamidomethylation	F2
77	T.ASVNC(+57.02)AKK(-1.03)IVSDGNGMNAWVAWR.N	90	112	Carbamidomethylation	F2
78	I.VSDGNGMNAWVAWR.N	99	112		F2
79	A.WRNRC(+57.02)K(+145.02)GTDVQAWIR.G	111	125	Carbamidomethylation; 3-(carbamidomethylthio)propanoyl	F2
80	N.R(+42.01)C(+57.02)KGTDVQAWIR.G	114	125	Acetylation (N-term); Carbamidomethylation;	F1
81	N.RC(+57.02)K(+117.02)GTDVQAWIR.G	114	125	Carbamidomethylation; N-Homocysteine thiolactone	F1
82	N.RC(+57.02)KGT(+59.02)DVQAWIR.G	114	125	Carbamidomethylation; Aminoethylcysteine	F1
83	R.C(+57.02)KGTDVQAWIRG.C	115	126	Carbamidomethylation	F1, F3
84	R.C(+57.02)KGTDVQ(-18.01).A	115	121	Carbamidomethylation,dehydratation	F3
85	G.T(+59.02)DVQAWIR.G	118	125	Aminoethylcysteine	F1

3. Supplementary References

1. Lyon, D. K. *et al.* Highly oxidation resistant inorganic-porphyrin analog polyoxometalate oxidation catalysts. 1. The synthesis and characterization of aqueous-soluble potassium salts of .a2-P2W17O61(Mn+.OH2)(n-10) and organic solvent soluble tetra-n-butylammonium salts a2-P2W 17061(Mn+.Br)(n-l l) (M = Mn3+, Fe3+, Co2+, Ni2+, Cu2+). *J Am Chem Soc* **113**, 7209–7221 (1991).

2. Grant, K. & Kassai, M. Major Advances in the Hydrolysis of Peptides and Proteins by Metal Ions and Complexes. *Curr Org Chem* **10**, 1035–1049 (2006).

3. Gómez Ruiz, B., Roux, S., Courtois, F. & Bonazzi, C. Spectrophotometric method for fast quantification of ascorbic acid and dehydroascorbic acid in simple matrix for kinetics measurements. *Food Chem* **211**, 583–589 (2016).

4. Qin, P., Su, B. & Liu, R. Probing the binding of two fluoroquinolones to lysozyme: A combined spectroscopic and docking study. *Mol Biosyst* **8**, 1222–1229 (2012).

5. Tanaka, F., Forster, L. S., Pal, P. K. & Rupley, J. A. The Circular Dichroism of Lysozyme. *J Biol Chem* **250**, 6977–6982 (1975).

6. Su, J. *et al.* Effect of tannic acid on lysozyme activity through intermolecular noncovalent binding. *J Agric Food Res* **1**, 100004 (2019).

7. Li, D., Ji, B. & Jin, J. Spectrophotometric studies on the binding of Vitamin C to lysozyme and bovine liver catalase. *J Lumin* **128**, 1399–1406 (2008).

8. Zhou, P. *et al.* Generation of hydrogen peroxide and hydroxyl radical resulting from oxygen-dependent oxidation of l-ascorbic acid via copper redox-catalyzed reactions. *RSC Adv* **6**, 38541–38547 (2016).

9. FİLİZ, İMER., ALDEMİR, E., KILIÇ, H., SONMEZOĞLU, İ. & APAK, R. The Protective Effect of Amino Acids on the Copper(II)−Catalyzed Autoxidation of Ascorbic Acid. *Int J Food Prop* **16**, 46–53 (2008).

10. Törnvall, U. Pinpointing oxidative modifications in proteins - Recent advances in analytical methods. *Analytical Methods* **2**, 1638–1650 (2010).

11. Ok Kang, J., Chan, P. C. & Kesner, L. Peroxidation of lysozyme treated with Cu(II) and hydrogen peroxide. *Inorganica Chim Acta* **107**, 253–258 (1985).

12. Permentier, H. P. & Bruins, A. P. Electrochemical oxidation and cleavage of proteins with on-line mass spectrometric detection: Development of an instrumental alternative to enzymatic protein digestion. *Journal of the American Society for Mass Spectrometry* vol. 15 1707–1716 Preprint at https://doi.org/10.1016/j.jasms.2004.09.003 (2004).

13. Marques, E. F., Medeiros, M. H. G. & Di Mascio, P. Lysozyme oxidation by singlet molecular oxygen: Peptide characterization using [18O]-labeling oxygen and nLC-MS/MS. *Journal of Mass Spectrometry* **52**, 739–751 (2017).

14. Stroobants, K. *et al.* Polyoxometalates as a novel class of artificial proteases: Selective hydrolysis of lysozyme under physiological pH and temperature promoted by a cerium(IV) Keggin-type polyoxometalate. *Chemistry - A European Journal* **19**, 2848–2858 (2013).

15. Vandebroek, L. *et al.* Protein-Assisted Formation and Stabilization of Catalytically Active Polyoxometalate Species. *Chemistry - A European Journal* **24**, 10099–10108 (2018).

16. Sereikaite, J. *et al.* Protein scission by metal ion-ascorbate system. *Protein Journal* **25**, 369–378 (2006).

17. Uehara, H., Luo, S., Aryal, B., Levine, R. L. & Rao, V. A. Distinct oxidative cleavage and modification of bovine [Cu– Zn]-SOD by an ascorbic acid/Cu(II) system: Identification of novel copper binding site on SOD molecule. *Free Radic Biol Med* **94**, 161–173 (2016).

18. Alqarni, M. H., Muharram, M. M., Alshahrani, S. M. & Labrou, N. E. Copper-induced oxidative cleavage of glutathione transferase F1-1 from Zea mays. *Int J Biol Macromol* **128**, 493–498 (2019).

19. Bridgewater, J. D. & Vachet, R. W. Metal-catalyzed oxidation reactions and mass spectrometry: The roles of ascorbate and different oxidizing agents in determining Cu–protein-binding sites. *Anal Biochem* **341**, 122–130 (2005).

20. Guilloreau, L., Combalbert, S., Sournia-Saquet, A., Mazarguil, H. & Faller, P. Redox Chemistry of Copper–Amyloid-β: The Generation of Hydroxyl Radical in the Presence of Ascorbate is Linked to Redox-Potentials and Aggregation State. *ChemBioChem* **8**, 1317–1325 (2007).

21. Singh, R. K. *et al.* Detection and characterization of a novel copper-dependent intermediate in a lytic polysaccharide monooxygenase. *chem.Eur.J.* **26**, 454–463 (2020).

22. Tadolini, B. & Cabrini, L. The influence of pH on OH scavenger inhibition of damage to deoxyribose by Fenton reaction. *Mol Cell Biochem* **94**, 97–104 (1990).

23. Galano, A. & Alvarez-Idaboy, J. R. Glutathione: Mechanism and kinetics of its non-enzymatic defense action against free radicals. *RSC Adv* **1**, 1763–1771 (2011).

24. Whiteman, M. & Halliwell, B. Thiourea and dimethylthiourea inhibit peroxynitrite-dependent damage: Nonspecificity as hydroxyl radical scavengers. *Free Radic Biol Med* **22**, 1309–1312 (1997).

25. Zhou, P. *et al.* Generation of hydrogen peroxide and hydroxyl radical resulting from oxygen-dependent oxidation of l-ascorbic acid via copper redox-catalyzed reactions. *RSC Adv* **6**, 38541–38547 (2016).

26. Methenitis, C., Skounas, S., Pneumatikakis, G. & Morcellet, M. Kinetic studies and mechanism of hydrogen peroxide catalytic decomposition by cu(II) Complexes with polyelectrolytes derived from l-alanine and glycylglycine. *Bioinorg Chem Appl* **2010**, (2010).

27. Perez-Benito, J. F. Reaction pathways in the decomposition of hydrogen peroxide catalyzed by copper(II). *J Inorg Biochem* **98**, 430–438 (2004).

28. Koppenol, W. H. Chapter 1 Chemistry of iron and copper in radical reactions. *New Comprehensive Biochemistry* **28**, 3–24 (1994).

29. Kim, B., Jeong, D., Ohta, T. & Cho, J. Nucleophilic reactivity of a copper(II)-hydroperoxo complex. *Commun Chem* **2**, 1–6 (2019).

30. Su, X. F., Guan, W., Yan, L. K. & Su, Z. M. Tricopper-polyoxometalate catalysts for water oxidation: Redox-inertness of copper center. *J Catal* **381**, 402–407 (2020).

31. Cheignon, C., Collin, F., Faller, P. & Hureau, C. Is ascorbate Dr Jekyll or Mr Hyde in the Cu(Aβ) mediated oxidative stress linked to Alzheimer's disease? *Dalton Transactions* **45**, 12627–12631 (2016).

32. Shevchenko, A., Tomas, H., Havli, J., Olsen, J. v & Mann, M. In-gel digestion for mass spectrometric characterization of proteins and proteomes. *Nat Protoc* **1**, 2856–2860 (2006).

33. Smiljanic, K. *et al.* In-depth quantitative profiling of post-translational modifications of Timothy grass pollen allergome in relation to environmental oxidative stress. *Environ Int* **126**, 644–658 (2019).

34. Hawkins, C. L. & Davies, M. J. Detection, identification, and quantification of oxidative protein modifications. *Journal of Biological Chemistry* **294**, 19683–19708 (2019).
