# Supplementary material for: Regioselective protein oxidative cleavage enabled by enzyme-like recognition of an inorganic metal oxo cluster ligand
Source: Nat Commun. 2023 Jan 30;14:486. doi: 10.1038/s41467-023-36085-z (PMC9887005; doi:10.1038/s41467-023-36085-z)
Supplement: Supplementary file 3 — Reporting Summary [file 41467_2023_36085_MOESM3_ESM.pdf]

nature portfolio

Corresponding author(s): Tatjana N. Parac-Vogt

Last updated by author(s): Jan 10, 2023

Reporting Summary

Nature Portfolio wishes to improve the reproducibility of the work that we publish. This form provides structure for consistency and transparency in reporting. For further information on Nature Portfolio policies, see our Editorial Policies and the Editorial Policy Checklist.

Statistics

For all statistical analyses, confirm that the following items are present in the figure legend, table legend, main text, or Methods section.

n/a	Confirmed	
☐	☒	The exact sample size (n) for each experimental group/condition, given as a discrete number and unit of measurement
☐	☒	A statement on whether measurements were taken from distinct samples or whether the same sample was measured repeatedly
☒	☐	The statistical test(s) used AND whether they are one- or two-sided<br>*Only common tests should be described solely by name; describe more complex techniques in the Methods section.*
☒	☐	A description of all covariates tested
☒	☐	A description of any assumptions or corrections, such as tests of normality and adjustment for multiple comparisons
☒	☐	A full description of the statistical parameters including central tendency (e.g. means) or other basic estimates (e.g. regression coefficient) AND variation (e.g. standard deviation) or associated estimates of uncertainty (e.g. confidence intervals)
☒	☐	For null hypothesis testing, the test statistic (e.g. F, t, r) with confidence intervals, effect sizes, degrees of freedom and P value noted<br>*Give P values as exact values whenever suitable.*
☒	☐	For Bayesian analysis, information on the choice of priors and Markov chain Monte Carlo settings
☒	☐	For hierarchical and complex designs, identification of the appropriate level for tests and full reporting of outcomes
☒	☐	Estimates of effect sizes (e.g. Cohen's d, Pearson's r), indicating how they were calculated

Our web collection on statistics for biologists contains articles on many of the points above.

Software and code

Policy information about availability of computer code

Data collection	Gel electrophoresis: Gel Doc EZ imager (Bio-Rad, Hercules, CA).<br>Mass spectrometry: Orbitrap Exploris 240 (Thermo Fisher Scientific, Bremen, Germany).<br>Fluorescence spectroscopy: FS-920P spectrofluorimeter (Edinburgh Instruments, Livingston, UK).<br>UV-Vis: Varian Cary 5000 spectrophotometer<br>EPR: ESP 300E<br>NMR: Bruker Avance 400 spectrometer
Data analysis	Gel electrophoresis: ImageLab version 6.0.0.<br>Mass spectrometry: PEAKS Studio X Pro software program (Bioinformatics Solutions Inc., Waterloo, ON, Canada).<br>Fluorescence spectroscopy: F980 version 1.4.3. and Origin 2018b software<br>UV-Vis: Cary WinUV version 3.00(182) and Origin 2018b software<br>EPR: Origin 2018b software<br>NMR: Topspin 4.0.6<br>Protein 3D structure: PyMOL version 2.5.4.

For manuscripts utilizing custom algorithms or software that are central to the research but not yet described in published literature, software must be made available to editors and reviewers. We strongly encourage code deposition in a community repository (e.g. GitHub). See the Nature Portfolio guidelines for submitting code & software for further information.

Data

Policy information about availability of data

All manuscripts must include a data availability statement. This statement should provide the following information, where applicable:

- Accession codes, unique identifiers, or web links for publicly available datasets
- A description of any restrictions on data availability
- For clinical datasets or third party data, please ensure that the statement adheres to our policy

The authors declare that the data supporting the findings of this study are available within the article and its Supplementary Information file; source data, including the uncropped SDS-PAGE gels, is provided with the paper as Source Data file. The mass spectrometry proteomics data generated in this study have been deposited to the ProteomeXchange Consortium via the PRIDE partner repository with the dataset identifier PXD037983

Human research participants

Policy information about studies involving human research participants and Sex and Gender in Research.

Reporting on sex and gender	N/A
Population characteristics	N/A
Recruitment	N/A
Ethics oversight	N/A

Note that full information on the approval of the study protocol must also be provided in the manuscript.

Field-specific reporting

Please select the one below that is the best fit for your research. If you are not sure, read the appropriate sections before making your selection.

[x] Life sciences [ ] Behavioural & social sciences [ ] Ecological, evolutionary & environmental sciences

For a reference copy of the document with all sections, see nature.com/documents/nr-reporting-summary-flat.pdf

Life sciences study design

All studies must disclose on these points even when the disclosure is negative.

Sample size	Sample size was determined based on similar studies in the field.
Data exclusions	No data exclusions were performed since this not applicable in this study.
Replication	To ensure reproducibility of this work all experiments have been repeated for at least 2-3 times.
Randomization	Samples were treated with specific conditions. This study does not contain patient or animal samples. It involves specific treatment of commercially acquired protein samples, thus randomization is not in relevant for this study.
Blinding	Protein samples needed to be treated under specific conditions, thus following common practice in the field blinding was not used.

Reporting for specific materials, systems and methods

We require information from authors about some types of materials, experimental systems and methods used in many studies. Here, indicate whether each material, system or method listed is relevant to your study. If you are not sure if a list item applies to your research, read the appropriate section before selecting a response.

Materials & experimental systems

n/a	Involved in the study	
☒	☐	Antibodies
☒	☐	Eukaryotic cell lines
☒	☐	Palaeontology and archaeology
☒	☐	Animals and other organisms
☒	☐	Clinical data
☒	☐	Dual use research of concern

Methods

n/a	Involved in the study	
☒	☐	ChIP-seq
☒	☐	Flow cytometry
☒	☐	MRI-based neuroimaging
